# From Smart Hydrogel Design to 4D-Printed Scaffolds: Emerging Paradigms in Precision Drug Delivery and Regenerative Wound Therapy

**DOI:** 10.3390/gels12050389

**Published:** 2026-05-01

**Authors:** Mariana Chelu, José María Calderón Moreno, Monica Popa

**Affiliations:** Institute of Physical Chemistry—Ilie Murgulescu, Romanian Academy, 060021 Bucharest, Romania; mchelu@icf.ro

**Keywords:** 3D/4D printing, smart hydrogels, natural/smart materials, stimuli-responsive polymers, drug delivery, wound healing

## Abstract

Smart hydrogel systems with stimuli-responsive properties are increasingly being investigated in combination with advanced additive manufacturing techniques for targeted drug delivery and wound healing in regenerative medicine; however, their clinical translation remains limited by challenges related to material performance, design complexity, and manufacturing scalability. This review analyzes recent developments in smart hydrogel design and 4D-printed scaffolds, with emphasis on programmable and stimuli-responsive architectures. The literature is selectively evaluated based on relevance to (i) hydrogel structure–property relationships, (ii) 3D/4D printing strategies, and (iii) demonstrated performance in drug delivery and wound healing applications. The analysis highlights design approaches enabling spatiotemporal control of drug release and dynamic scaffold behavior, while also examining how fabrication methods influence functional outcomes. Major limitations are critically assessed, including issues of reproducibility, mechanical stability, long-term performance, and the gap between experimental studies and clinical application. Challenges in defining and implementing 4D printing in biomedical contexts are discussed as well. Overall, this review identifies current design trade-offs, outlines priorities for improving reliability and translational potential, and synthesizes emerging trends in 3D and 4D printed hydrogel scaffolds for precision drug delivery and regenerative wound therapy.

## 1. Introduction

The ability to fabricate three-dimensional (3D) structures has significantly advanced modern fabrication technologies. Over the past few years, hydrogels have gained considerable attention as highly promising feedstock materials for 3D printing, particularly for the fabrication of hydrated constructs that closely mimic the extracellular matrix (ECM) of native tissues [1,2,3]. Since their initial application as contact lenses in biomedical practice, hydrogels have undergone substantial material and technological evolution enabling the development of complex and multifunctional architectures [4,5,6].

These advances have enabled the production of 3D-printed architectures incorporating diverse organic and inorganic components, living cells, and various bioactive molecules. Hydrogels have a wide range of biomedical applications that can generally be grouped into several major categories, including tissue engineering and regenerative medicine, three-dimensional cell culture and disease modeling, drug screening and toxicity evaluation, and the development of innovative biomedical devices and drug delivery platforms [7,8,9,10,11].

Although there has been considerable progress in these areas, several limitations persist that prevent the full use of hydrogels in 3D printing technologies [12,13,14]. Addressing these limitations is essential to further expansion of their practical applications. The challenges include enhancing printing precision and structural intricacy, maintaining and improving cell viability and biological functionality, increasing cost-effectiveness and broader accessibility, and overcoming ethical and regulatory barriers associated with their clinical implementation [15,16].

Recent advancements in smart hydrogel technologies have led to the development of materials capable of dynamically responding to biological stimuli, enabling the efficient delivery of therapeutic agents for wound repair [17,18,19,20]. These stimuli-responsive hydrogels have significantly improved the safety and effectiveness of therapeutic compound delivery, contributing to accelerated and more reliable wound healing processes [21]. Innovations in smart hydrogel materials have transformed drug delivery strategies by enabling the controlled and localized release of bioactive agents directly at wound sites [22]. Progress in the design and engineering of these advanced hydrogel systems has also enhanced their capacity for the encapsulation of therapeutic molecules and their stimuli-responsive release, supporting improved outcomes in regenerative wound therapy [23,24,25].

**Figure 1:**
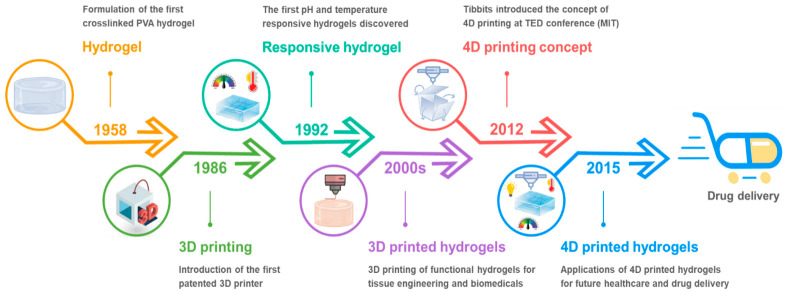
Overview of the development of 4D printed smart hydrogels for drug delivery applications. Reprinted from [34].

Starting in 2013, four-dimensional (4D) printing has integrated stimulus-responsive materials with three-dimensional (3D) printing technologies [26]. The fourth dimension, time, enables printed scaffolds to undergo programmable shape transformations in response to external stimuli, offering promising approaches for minimally invasive surgical applications [27,28]. Recent developments in smart hydrogel-based bioinks have facilitated the fabrication of 4D-printed scaffolds capable of controlled deformation [29,30,31]. This advancement enables printed structures to undergo autonomous shape transformations in response to external stimuli such as light, heat, electricity, or magnetic fields, a process termed 4D printing [32]. Unlike conventional 3D printed hydrogels, 4D printed systems are dynamic and programmable. This allows them to interact with their environment to exhibit diverse outputs, including shape morphing, mechanical actuation, and biologically relevant responses. Consequently, 4D printed smart hydrogels have garnered significant attention in both academic research and industrial applications [33]. Figure 1 provides a concise overview of the evolution from early cross-linked polymer networks to 4D printed hydrogels for drug delivery purposes [34].

Despite the rapid progress in 3D and 4D printed hydrogel systems, a clearly defined research gap persists at the intersection of material design, functional performance, and clinical translation. The current literature often addresses hydrogel formulation, printing strategies, or biomedical applications in isolation, with limited integration of these aspects into a unified framework specifically tailored for targeted drug delivery and wound healing. Moreover, while numerous studies highlight stimuli-responsive and programmable behaviors, there remains insufficient critical comparison of how these advanced properties translate into therapeutic efficacy, spatial–temporal control of drug release, and long-term regenerative outcomes in clinically relevant settings. Some studies focus either on general hydrogel biofabrication, smart materials, or 4D printing concepts independently, without systematically evaluating their combined potential or comparing their performance with conventional treatment strategies. In addition, significant limitations continue to hinder progress, including challenges in achieving precise control over multi-material deposition, scalability of fabrication processes, reproducibility of complex architectures, and maintenance of cell viability and bioactivity during and after printing. Regulatory uncertainty, high production costs, and limited standardization further restrict clinical applications. Importantly, there is a lack of comprehensive analyses comparing different hydrogel systems in terms of their responsiveness, mechanical stability, degradation behavior, and drug delivery efficiency under physiologically relevant conditions.

Therefore, this review aims to bridge these gaps by providing a comparative and integrative assessment of 3D and 4D printed hydrogel scaffolds, with a specific focus on their application in targeted drug delivery and wound healing. By critically assessing recent advancements alongside existing limitations and positioning emerging technologies in relation to current clinical needs and benchmarks, this review advances beyond prior publications to provide a more coherent and current understanding of how smart hydrogel-based biofabrication can be effectively translated into next-generation regenerative therapies. We adopt a framework that links material properties, printing strategies, and functional outcomes, with particular emphasis on emerging design trade-offs and translational challenges. The purpose of this review is to identify and track emerging trends in the field of 4D-printed smart hydrogel scaffolds, with particular emphasis on evolving paradigms in precision drug delivery and regenerative wound therapy, as well as to provide critical insights into the progress of adaptive and clinically responsive hydrogel systems.

## 2. Smart Hydrogel Design for Drug Delivery and Wound Healing

In recent decades, smart hydrogel-based scaffolds have emerged as promising biomaterials in biomedical research, particularly within the fields of drug delivery, wound management, and regenerative medicine [35,36].

Hydrogels consist of highly hydrated three-dimensional polymer networks capable of absorbing and retaining large volumes of water while maintaining structural integrity. They can be synthesized from natural, synthetic, or hybrid polymeric materials and are typically formed through either physical interactions or covalent crosslinking [37,38,39]. Due to their high water content, permeability, and structural resemblance to the extracellular matrix, hydrogels provide a favorable microenvironment for both drug encapsulation and cellular activity [40,41,42,43]. In 3D hydrogel-based scaffolds, the porous network functions not only as a reservoir for therapeutic agents but also as a supportive framework that promotes cell adhesion, proliferation, and tissue regeneration [44]. From a mechanistic perspective, the performance of hydrogel scaffolds is governed by the interplay between polymer network architecture, water–polymer interactions, and mass transport phenomena [35,36]. Drug loading and release are primarily controlled by diffusion through water-filled pores, polymer relaxation (swelling/deswelling), and in degradable systems, network erosion [36,37,40]. The mesh size, typically in the range of 5–100 nm for most biomedical hydrogels, is a critical parameter that determines whether drug transport is diffusion-limited or sterically hindered [35,36]. For small molecules (Mw < 500 Da), release is often Fickian and rapid, whereas for macromolecules such as proteins (10–100 kDa) transport becomes increasingly restricted and may require network degradation or swelling-induced expansion. In stimuli-responsive systems, external triggers (e.g., pH, temperature, enzymes) alter polymer conformation or crosslink density, thereby modulating mesh size and enabling on-demand drug release [45].

Recently, the development of 4D hydrogel systems—materials capable of undergoing controlled time-dependent changes in response to environmental stimuli such as temperature, pH, or biochemical signals—has enabled the design of dynamic scaffolds that can adapt their shape, structure, or drug-release behavior after implantation [45,46]. Mechanistically, these transformations arise from reversible physicochemical transitions (e.g., LCST-driven collapse in thermoresponsive polymers or ionization changes in pH-sensitive networks), which directly influence swelling kinetics, diffusivity, and mechanical properties.

Another important advantage of hydrogel scaffolds is their capacity to incorporate and deliver a wide range of therapeutic compounds, including small-molecule drugs, proteins, growth factors, and nucleic acids [47,48,49]. Hydrogels used in scaffold fabrication are commonly classified into three categories: natural, synthetic, and semi-synthetic [50,51]. Natural hydrogels are derived from biopolymers such as chitosan, alginate, fibrin, gelatin, and hyaluronic acid, all of which offer excellent biocompatibility and biological functionality [52,53]. Synthetic hydrogels, including those based on poly(ethylene glycol) (PEG) or poly(vinyl alcohol) (PVA), provide greater control over mechanical properties, degradation kinetics, and structural stability [54]. Semi-synthetic hydrogels combine the beneficial characteristics of both groups; for example, gelatin methacryloyl (GelMA) is produced by chemically modifying gelatin with methacryloyl groups, enabling improved crosslinking and mechanical strength while maintaining the bioactive features of the original polymer [55,56]. Hydrogels based on cellulose derivatives, such as methylcellulose (MC), are also widely used in tissue engineering and regenerative medicine due to their biocompatibility, biodegradability, non-toxicity, and tunable physicochemical characteristics. As semisynthetic biopolymers, they offer a practical alternative to water-insoluble natural cellulose, making them suitable for scaffold fabrication, drug delivery platform design, and serving as bioinks for 3D bioprinting [57].

Table 1 summarizes several basic characteristics of natural and synthetic polymers that must be taken into account during the design and fabrication of smart hydrogel systems.

The functional performance of hydrogel scaffolds is intrinsically linked to their structure–property relationships, particularly network density, crosslinking type, and polymer composition [35,36,38,40]. Crosslinking mechanisms can be broadly divided into:

(i) Physical crosslinking (ionic interactions, hydrogen bonding, hydrophobic interactions) typically results in reversible, shear-thinning, and injectable hydrogels with lower mechanical strength (elastic modulus ~0.1–10 kPa) and faster drug release due to larger and more dynamic mesh sizes.
(ii) Chemical (covalent) crosslinking (photo-polymerization, click chemistry, enzymatic reactions) produces more stable networks with higher stiffness (10–1000 kPa), reduced swelling, and slower drug release with more control.
(iii) Dynamic covalent and supramolecular crosslinking enables reversible bond formation combining structural stability with stimuli responsiveness, which is particularly relevant for 4D systems.

The crosslink density directly influences main properties such as swelling ratio, mesh size, and diffusion coefficient. Quantitatively, increasing crosslink density reduces the mesh size and diffusivity, often following an exponential relationship. For example, alginate hydrogels (ionic crosslinks) have a mesh size of ~20–80 nm, rapid release (50–80% drug release in 24 h for small molecules) but limited mechanical robustness. In comparison, PEG-based hydrogels (covalently crosslinks) show a mesh size of ~5–20 nm and slower release profiles (20–50% over several days) with tunable degradation. Meanwhile, Gel-MA-type hydrogels (photocrosslinked semisynthetics) exhibit an intermediate mesh size (~10–50 nm), balanced mechanical strength (10–100 kPa), and sustained release over days to weeks.

The type of crosslinking and architecture of the network are characteristics that directly determine drug release performance through three main mechanisms [35,36,37,38,40]:

(i) Diffusion-controlled release is dominant in highly swollen and weakly crosslinked hydrogels.
(ii) Swelling-controlled release is observed in stimuli-responsive systems, where environmental changes modify the expansion of the network.
(iii) Degradation-controlled release is typical in enzymatically or hydrolytically degradable hydrogels, allowing for long-term release (weeks to months).

For example, tightly crosslinked PEG systems may achieve prolonged release of proteins over 2–4 weeks, but may limit initial loading efficiency. In contrast, loosely cross-linked natural hydrogels allow for high loading, but suffer from burst release (>60% within the first 12–24 h).

A comparative evaluation of hydrogel systems highlights the inherent trade-offs between the main properties of the materials (Table 2).

These comparisons highlight some design trade-offs: (i) increasing mechanical strength (through higher crosslink density) often reduces drug diffusivity and cellular infiltration; (ii) improving bioactivity (natural polymers) may compromise structural stability and reproducibility; and (iii) improving reactivity (4D systems) may introduce complexity in manufacturing and variability in performance.

Despite significant advances, current hydrogel systems often fail to simultaneously optimize mechanical integrity, biological functionality, and precise drug delivery control. Many studies prioritize individual properties (e.g., responsiveness or biocompatibility) without fully addressing their interdependence under physiological conditions. Furthermore, quantitative standardization across studies remains limited, making direct comparison of performance metrics challenging. As a result, the rational design of hydrogel scaffolds still requires a more integrated mechanism-driven approach that systematically balances these competing parameters.

In regenerative medicine, 3D and 4D hydrogel-based scaffolds are increasingly recognized as multifunctional therapeutic platforms capable of integrating structural support, controlled drug delivery, and biological signaling within a single system [90,91,92,93]. These scaffolds can be designed to deliver therapeutic agents directly to injured or diseased tissues, thereby enhancing wound healing, reducing infection, and promoting tissue regeneration [94,95]. Furthermore, the adaptability of hydrogel scaffolds allows them to be applied through various administration routes, including topical and transdermal treatments for wound care, localized implantation for tissue repair, and other targeted delivery strategies [96,97,98]. As a result, 3D and 4D hydrogel architectures represent a promising and rapidly-developing approach for the next generation of biomaterials used in advanced drug delivery and regenerative medicine applications.

## 3. Advanced Methods for 3D/4D Printing of Hydrogel Scaffolds

Advanced 3D and 4D printing technologies are increasingly used for the fabrication of hydrogel-based scaffolds in regenerative medicine. These approaches enable controlled spatial deposition of hydrogel bioinks, facilitating the design of architectures that support tissue regeneration while allowing incorporation of therapeutic agents for localized drug delivery [99,100,101,102]. In 4D printing, the use of stimuli-responsive hydrogels introduces time-dependent behavior, by which scaffolds undergo structural or physicochemical changes in response to environmental cues [103,104,105]. Such dynamic properties can influence drug release profiles and scaffold–tissue interactions. However, their performance remains dependent on factors such as response kinetics, material stability, and reproducibility, which continue to limit translation into clinical applications.

### 3.1. 3D Printing Technologies for Hydrogel Scaffold Fabrication

Since its introduction in 1986, three-dimensional (3D) printing has enabled the direct fabrication of structures from computer-aided design models, reducing material waste and production time [106,107]. Over time, its application has expanded into the biomedical field, where it supports the fabrication of patient-specific scaffolds with controlled geometries and architectures [108,109,110]. In particular, 3D printing provides a means to engineer hydrogel-based constructs with tunable structural features relevant to drug delivery and wound healing [111,112].

**Figure 2:**
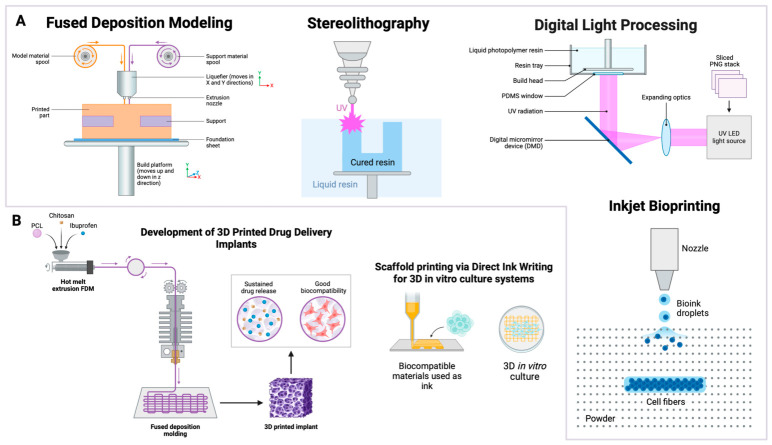
Overview of common 3D printing techniques and applications. (**A**) FDM deposits thermoplastic layer-by-layer; SLA and DLP cure photopolymer resins using UV light or projected images; inkjet bioprinting prints bioinks and cells for tissue engineering. (**B**) Applications include FDM-based drug delivery implants and DIW-printed scaffolds for in vitro culture systems. Reprinted from [113].

Several printing modalities are used, including material extrusion, vat polymerization, powder bed fusion, material jetting, binder jetting, directed energy deposition, and sheet lamination [113]. Figure 2 summarizes commonly used techniques and their typical applications [113].

**Figure 3:**
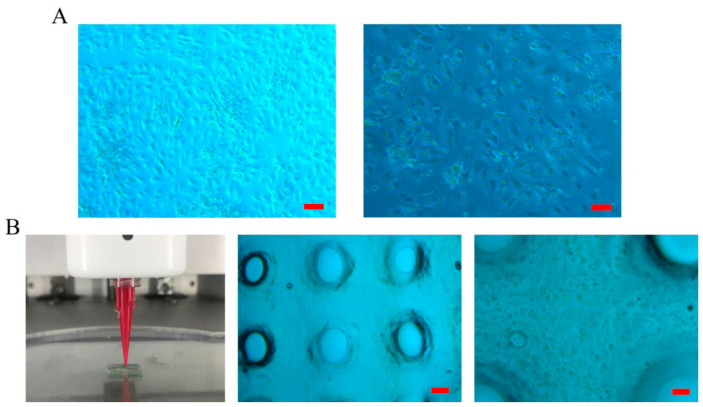
Morphology of primary human parathyroid cells and 3D-bioprinted constructs. (**A**) Morphology of isolated primary human parathyroid cells. (**B**) 3D bioprinting process and distribution and morphology of cells within the printed scaffold. Scale bars: 100 μm (**A left**,**B middle**) and 40 μm (**A right**,**B right**) Reprinted from [114].

As an important method in tissue engineering, 3D bioprinting enables the precise creation of functional tissues and organs in the laboratory. It enables the precise arrangement of living cells to create complex biological structures, supports personalized medicine, and advances transplantation research towards clinical use. Among the complications frequently encountered after thyroid surgery, hypocalcemia caused by hypoparathyroidism presents certain long-term risks such as osteoporosis, cardiac dysfunction, and renal failure, which makes lifelong calcium regulation absolutely essential. A recent study utilizing extrusion-based 3D bioprinting (e.g., direct ink writing) investigated the fabrication of human parathyroid tissue constructs using an alginate-based hydrogel (Figure 3) [114]. This approach enables the deposition of cell-laden bioinks under mild conditions, preserving cell viability and allowing the incorporation of functional endocrine cells within defined architectures. Preliminary results showed functional parathyroid cells expressing the surface calcium-sensing receptor (CaSR) for up to three weeks. However, by three months scaffold pores had become filled with calcified and fibrotic tissue; parathyroid hormone (PTH) expression was lost, indicating a decline in graft function. These outcomes highlight significant limitations associated with extrusion-based bioprinting of alginate hydrogels, particularly in terms of long-term structural stability, mass transport, and host response. While short-term hormonal activity was achieved, the results underscore the need to optimize bioink composition and crosslinking strategies in order to improve scaffold biocompatibility and sustain functionality to enhance the therapeutic potential of bioprinted parathyroid tissue.

Finally, the above study is subject to several limitations, and further research targeting more quantitative functional analyses, improved biomaterials, and immunomodulatory approaches is needed to support clinical translation.

**Figure 4:**
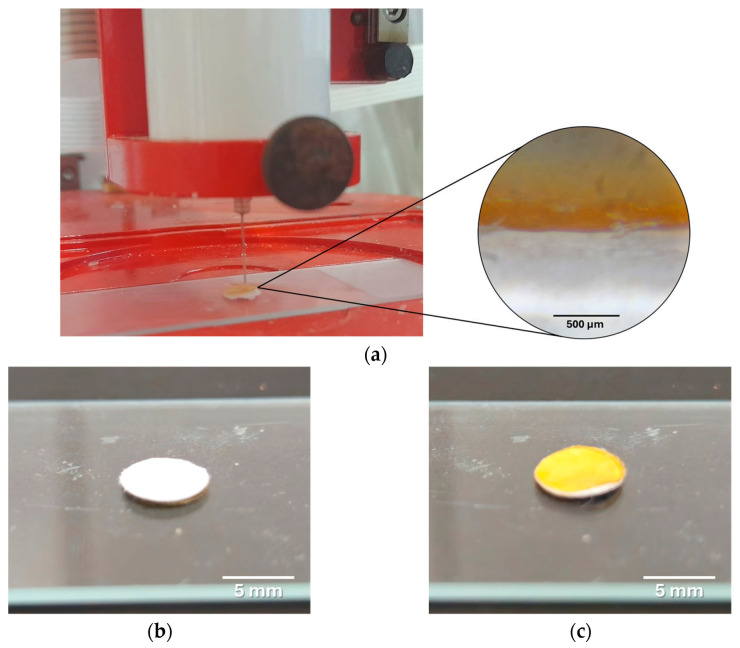
The 3D-printed hybrid scaffold. (**a**) Printing process with magnified interface between PCL/Lap and chitosan/collagen/DBP hydrogel layers. (**b**) PCL/Lap scaffold alone. (**c**) scaffold with hydrogel layer applied. Reprinted from [115].

A new study employed a multi-material 3D printing strategy to fabricate a hybrid scaffold (Figure 4), combining extrusion-based deposition of poly(ε-caprolactone)/nanosized synthetic smectic clay (PCL/Lap) nanocomposites for mechanical reinforcement with hydrogel printing of a chitosan/collagen/demineralized bone powder matrix for enhanced bioactivity and fluid retention [115]. This approach allows for spatial control over scaffold composition, enabling the integration of mechanically robust and biologically active phases within a single construct. This is particularly relevant for engineering complex interfaces such as the subchondral bone–calcified cartilage region. The primary challenge of this study lies in addressing the inherent trade-off between mechanical strength and bioactivity when both are required for effective tissue regeneration. However, this multi-material printing strategy also presents challenges such as interfacial bonding between phases, differences in degradation rates, and the need to balance printability with biological functionality. These factors remain critical considerations in optimizing scaffold performance for osteochondral applications.

**Figure 5:**
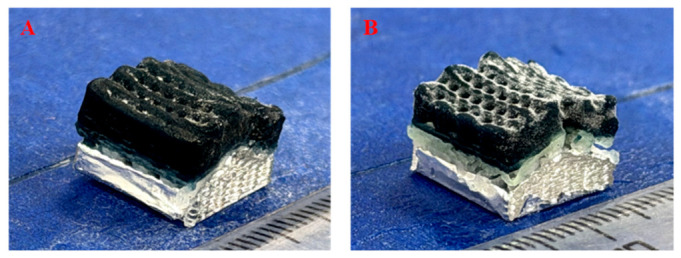
Macroscopic view and hydroxyapatite (HA) distribution in scaffolds. (**A**) Continuously graded HA (CG-HA). (**B**) Stepwise HA (S-HA). Reprinted from [116].

A dual-channel extrusion-based 3D printing strategy was used to fabricate a biomimetic spatially-curved cartilage scaffold with a continuous hydroxyapatite (HA) gradient, mimicking the transition from calcified to non-calcified cartilage (Figure 5) [116]. This approach enables precise spatial control over composition and architecture, allowing the generation of functionally graded structures that are difficult to achieve with conventional single-material printing techniques. The engineered gradient and curved geometry contributed to improved compressive strength and stable swelling and degradation behavior. However, such multi-channel printing strategies require careful optimization of rheological properties to ensure synchronized deposition and structural fidelity, particularly when combining materials with distinct viscosities and crosslinking mechanisms. While in vitro results demonstrated good cytocompatibility and supported bone marrow mesenchymal stem cell (BMSC) adhesion, proliferation, and cartilage matrix synthesis, maintaining gradient stability and reproducibility during long-term use remains an essential challenge for translation. Although this design enables the fabrication of tailored architectures for bone or osteochondral repair, the use of precisely controlled continuous compositional gradients to achieve a gradual transition from calcified to non-calcified cartilage is not a widely applied strategy.

In summary, in the studies presented above, the main advances brought by 3D printing have allowed for (i) precise spatial control over the distribution of cells and materials; (ii) integrated mechanical and biological functionalities to mimic native tissue environments; and (iii) short-term functional viability (parathyroid) and cytocompatibility (cartilage/osteochondral scaffolds).

Several limitations remain to be addressed, including (i) long-term viability and hormonal activity are limited by scaffold degradation and fibrosis (parathyroid); (ii) mechanical stability may still be questioned under in vivo loading for osteochondral scaffolds; and (iii) gradient scaffolds, although promising, require further in vivo validation for functional integration and durability.

Overall, while 3D printing enables highly biomimetic tissue constructs, persistent challenges remain regarding long-term functionality, scaffold biocompatibility, and mechanical strength under physiological conditions.

### 3.2. 4D Printing in Smart Hydrogel Systems

Four-dimensional (4D) printing extends additive manufacturing by enabling the fabrication of structures that undergo time-dependent changes in shape, properties, or function in response to external stimuli. The 4D printing concept introduces time as an additional design parameter in printed systems [117]. In biomedical applications, 4D bioprinting may incorporate cell-laden bioinks to generate constructs with dynamic behavior [118]. Two main approaches are typically distinguished: (i) preprogrammed material transformation, in which stimuli-responsive polymers or hydrogels undergo controlled shape or property changes under defined conditions; and (ii) in vivo transformation where printed scaffolds evolve post-implantation, for example through controlled degradation that supports tissue development [119].

Smart hydrogel systems that enable dynamic responses to environmental stimuli undergo volumetric or structural changes due to swelling, deflation, or phase transitions triggered by pH, temperature, light, or ionic strength. The underlying physicochemical mechanisms are based on the reversible reconfiguration of the polymer network, driven by hydrogen bonds, ionic interactions, and hydrophobic-hydrophilic equilibrium. The crosslink density and polymer chain mobility dictate the rate and extent of actuation. Swelling and deflation are central mechanisms, driven by osmotic pressure differences between the hydrogel and its environment. Temperature-sensitive hydrogels exploit the lower or upper critical solution temperature (LCST/UCST), where polymer–solvent interactions change, causing phase transitions. pH-sensitive hydrogels rely on ionizable groups that gain or lose charge, modifying electrostatic repulsion and network expansion. Light- or chemical-responsive hydrogels utilize photosensitive or redox-active components that change their conformation under the influence of specific stimuli. Hydrogen bonds, hydrophobic interactions, and ionic crosslinks dynamically reorganize the network, translating molecular-level responses into macroscopic shape changes. In addition, gradient crosslinking or anisotropic structures allow for directional actuation, enhancing the programmability of the hydrogel response. Incorporation of functional nanoparticles can enhance reactivity or introduce multi-stimulus control. By integrating material programming and kinetic control, 4D printing creates hydrogels capable of shape transformations over time. These hydrogels embed stimuli-responsive elements, allowing them to exhibit predictable deformation when exposed to external cues, which has the potential for dynamic biomedical applications. However, current 4D printing approaches also face significant challenges. For example, reproducibility is hindered by variations in hydrogel composition and printing conditions, while scalability remains limited due to the slow kinetics of shape evolution and constraints in multi-material fabrication. Furthermore, long-term stability under physiological conditions is not fully established, complicating clinical translation. Addressing these limitations will require standardized protocols, enhanced material design, and systematic evaluation of performance in relevant biological contexts.

The inherent smartness of the material in 4D printed products allows the state and function of the object to be programmed, eliminating the need for external devices or postprocessing methods. This not only shortens production time but can also facilitate the application process [120]. For instance, shape-transforming smart scaffolds which remain compact before implantation can enable minimally invasive procedures and subsequently self-assemble into complex structures through their dynamic response once inside the body [121,122]. Smart hydrogels are particularly well-suited for 4D bioprinting [123]. These water-swollen polymer networks respond to stimuli such as pH, temperature, light, magnetic fields, or mechanical stress [124,125,126]. Mechanical properties of hydrogels range from purely elastic to viscoelastic depending on crosslinking type and density, offering tunable performance for dynamic bioprinted systems [127,128,129]. By leveraging 3D printing advantages, hydrogels can be programmed for self-folding, self-welding, shape-memory, and controllable sol–gel transitions, enabling advanced tissue engineering applications including the fabrication of functional ocular, liver, and heart tissues [130,131,132,133].

**Figure 6:**
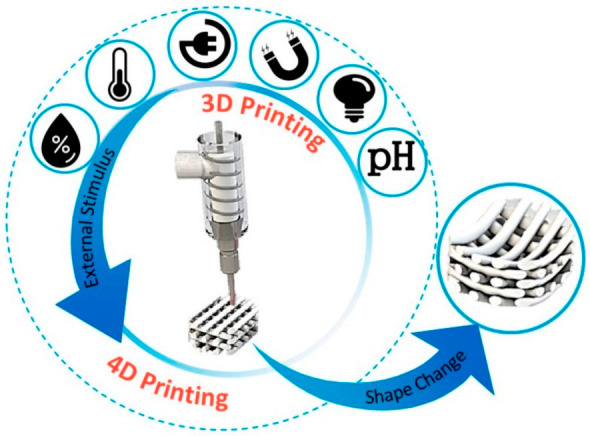
Diagram illustrating the application of a predefined stimulus to induce a targeted response in a 3D/4D bioprinted construct. Reprinted from [140].

Technologies for 3D and 4D bioprinting provide an innovative strategy, including for ocular tissue engineering. Through 3D bioprinting, highly accurate scaffolds can be fabricated to support corneal and retinal regeneration [134,135,136]. Meanwhile, 4D bioprinting utilizes smart materials that can adapt and respond to the biological environment, enabling the creation of dynamic structures that function effectively within living tissues [137,138]. Emerging applications of 4D bioprinting have demonstrated advancements in areas such as optic nerve regeneration and responsive corneal repair, offering promising possibilities for treating visual impairments [139]. Despite significant progress, challenges remain in achieving optimal biomaterial–tissue compatibility and effective neural integration. Additional barriers include material selection, biocompatibility concerns, high costs, and limited specialized expertise, which continue to hinder widespread clinical adoption. Figure 6 illustrates 4D printing, where 3D-printed structures change their shape, properties, or function over time or in response to stimuli.

### 3.3. Comparative Analysis of 3D and 4D Printing Techniques for Hydrogel Scaffolds

A critical comparison of advanced 3D and 4D bioprinting techniques reveals important trade-offs in terms of resolution, bioink compatibility, cell viability, and scalability, which are some of the essential parameters that determine their suitability for regenerative medicine applications. On the one hand, it should be taken into account that the printability of bioinks is fundamentally governed by their rheological properties, in particular the shear thinning behavior and the viscosity and gelation kinetics; collectively, these determine their ability to be deposited precisely while maintaining structural fidelity [111]. On the other hand, ideal bioinks should exhibit shear-thinning behavior in order to facilitate smooth extrusion under applied stress followed by rapid viscosity recovery to preserve the printed architecture. In addition, controlled gelation kinetics are essential to ensure post-deposition shape retention without compromising cell encapsulation or function.

In terms of printing resolution, vat polymerization techniques (e.g., stereolithography and digital light processing) achieve the highest spatial resolution (down to tens of micrometers), enabling the fabrication of highly intricate microarchitectures [109,111,113,121]. Material jetting also offers high precision, but is limited by bioink viscosity constraints [109,110,111,113]. In contrast, material extrusion, one of the most widely used methods, typically provides lower resolution (100–500 µm), although it remains sufficient for many tissue engineering applications [109,110,111,113]. In 4D printing these resolution limits are inherited from the underlying 3D printing technique, with additional complexity arising from the need to preserve the functionality of stimuli-responsive materials [122,123,124].

Regarding bioink compatibility, extrusion-based systems are the most versatile, accommodating a wide range of hydrogel formulations including high-viscosity and cell-laden bioinks. Vat polymerization requires photocrosslinkable bioinks, which restricts material selection and may involve photoinitiators with potential cytotoxicity. Material jetting demands low-viscosity bioinks, also limiting the range of usable hydrogels. Bioink selection becomes even more constrained in 4D printing, as materials must exhibit predictable and reproducible responsiveness to stimuli (e.g., thermo-responsive or pH-sensitive polymers), often narrowing the pool of clinically suitable candidates [121,122,123].

At the same time, another critical parameter is cell viability, which is strongly influenced by the printing mechanism [109,113,118]. Extrusion-based bioprinting can impose shear stress on encapsulated cells, potentially reducing viability, although optimization of nozzle diameter and pressure can mitigate these effects. Vat polymerization offers high cell viability under carefully controlled light exposure conditions, but carries risks of phototoxicity. Material jetting may expose cells to thermal or mechanical stress during droplet formation. In 4D systems, maintaining cell viability is further complicated by dynamic transformations of the scaffold, requiring that both the printing process and subsequent stimuli-induced changes remain cytocompatible over time [118,120,123].

From a scalability and clinical translation perspective, extrusion-based methods are currently the most accessible and scalable due to their relative simplicity, cost-effectiveness, and compatibility with a wide range of biomaterials. Vat polymerization, while precise, faces limitations in construct size and material diversity. More complex methods such as directed energy deposition are less commonly used in soft biomaterials. Although highly promising, 4D bioprinting remains at an early stage of development, with challenges related to reproducibility, manufacturing standardization, and regulatory approval. Additionally, the integration of time-dependent functionality introduces further complexity in quality control and long-term performance validation.

Overall, while 3D bioprinting provides robust and relatively mature platforms for hydrogel scaffold fabrication, 4D bioprinting offers transformative potential through dynamic stimuli-responsive behavior. However, this added functionality comes at the cost of increased material constraints, system complexity, and translational challenges. Future advancements will likely focus on hybrid approaches that balance high resolution, cytocompatibility, and programmable functionality to enable clinically viable smart scaffolds.

## 4. Hydrogel Scaffold Design for Drug Delivery and Wound Healing

The design of 3D and 4D hydrogel scaffolds is increasingly explored in regenerative medicine as a platform for controlled drug delivery and wound healing. These hydrated, biocompatible networks provide structural support while enabling the sustained release of therapeutic agents. Reported systems incorporate a wide range of cargos, including small molecules (e.g., doxorubicin, ibuprofen, dexamethasone), proteins and peptides (e.g., insulin, growth factors such as vascular endothelial growth factor, antibodies), nucleic acids (DNA, siRNA), and cells or biological components (e.g., stem cells, probiotic bacteria). In addition, antibacterial agents, antiseptics, and enzymes can be integrated into stimuli-responsive hydrogel systems [141].

While 3D hydrogels provide stable structural environments for cell growth and tissue regeneration, 4D hydrogels introduce dynamic, stimuli-responsive properties that allow the scaffold to change shape or function over time in response to physiological conditions [142,143]. This adaptability makes them particularly valuable for treating a wide range of conditions, including chronic wounds, burns, diabetic ulcers, tissue defects, and post-surgical injuries. Due to their tunable properties, minimal invasiveness, and compatibility with biological tissues, hydrogel scaffolds can benefit patients across all age groups, from pediatric tissue repair to regenerative therapies for age-related degenerative diseases, underscoring their growing importance in next-generation biomedical treatments.

### 4.1. Drug Delivery

Hydrogel scaffolds represent an advanced class of biomaterials for drug delivery in regenerative medicine, enabling precise spatial control, customizable architecture, and dynamic responsiveness to physiological environments. Through additive manufacturing techniques, these scaffolds can be engineered to mimic the native extracellular matrix while simultaneously acting as reservoirs for therapeutic agents that support tissue regeneration and repair [144]. One crucial aspect of these systems is drug encapsulation strategies, which allow bioactive molecules such as growth factors, antibiotics, anti-inflammatory drugs, or stem-cell–modulating agents to be incorporated directly into the hydrogel matrix [145,146,147,148,149,150,151].

Pharmacological modulation of stem cell signaling pathways has been developed for therapeutic applications. Encapsulation can be achieved through physical entrapment, covalent bonding, nanoparticle integration, or microcarrier incorporation, enabling protection of sensitive therapeutics from degradation and improving their stability within the biological environment [152]. Equally important are controlled and sustained release mechanisms, which ensure that therapeutic compounds are delivered over extended periods rather than through rapid burst release [153]. By tuning the hydrogel crosslinking density, polymer composition, porosity, and degradation rate, researchers can regulate the diffusion and degradation-driven release of encapsulated drugs, thereby maintaining therapeutic concentrations at the target site and reducing the need for repeated administration.

More advanced platforms incorporate multi-drug delivery systems in which different therapeutic agents are loaded into distinct compartments or layers of the scaffold [154]. This design allows for sequential or simultaneous release profiles, enabling combination therapies that may include antimicrobial agents to prevent infection, angiogenic factors to stimulate blood vessel formation, and regenerative molecules that promote cell proliferation and tissue repair.

The development of stimuli-triggered and targeted drug release further enhances the therapeutic potential of 3D and 4D printed hydrogels. These smart scaffolds can respond to environmental cues such as pH changes, temperature, enzymatic activity, mechanical stress, or light. In 4D systems, the scaffold can dynamically alter its structure or permeability over time, enabling on-demand drug release precisely when the biological environment requires it, which is particularly beneficial for wound healing, tissue engineering, and localized regenerative therapies. Finally, pharmacokinetic considerations play a crucial role in the design of hydrogel-based drug delivery scaffolds, as they enable the optimization of drug release profiles, distribution, and local bioavailability to ensure safe and effective therapeutic outcomes for patients across all age groups, including pediatric populations [155]. Parameters such as diffusion rates, scaffold degradation kinetics, drug–polymer interactions, and local tissue absorption influence the overall bioavailability and therapeutic efficacy of the delivered compounds [156]. Drug release from carriers can be quantitatively described using kinetic or mathematical models that capture encapsulation efficiency and temporal release profiles. Systems are broadly classified as diffusion-controlled (where Fickian transport dominates) or degradation-controlled (where polymer erosion governs release). Burst release and inter-individual in vivo variability complicate predictable dosing, necessitating incorporation of pharmacokinetic parameters to link carrier-level kinetics with systemic drug concentration. Rigorous modeling enables the optimization of carrier design and dosing strategies to achieve sustained and reproducible therapeutic effects. By tailoring scaffold geometry and material properties through 3D/4D printing technologies, it is possible to optimize drug distribution, prolong local retention, and minimize systemic side effects [157]. Together, these features make 3D and 4D printed hydrogel scaffolds highly promising platforms for localized, programmable, and patient-specific drug delivery in regenerative medicine, supporting improved treatment outcomes for a wide range of tissue repair and healing applications.

### 4.2. Wound Healing

Wound healing is a highly coordinated biological process that restores tissue integrity following injury. It typically proceeds through four overlapping phases: hemostasis, inflammation, proliferation, and remodeling [158,159]. Immediately after injury, the hemostatic phase is initiated by platelet aggregation and fibrin clot formation, which stabilize the wound site and provide a provisional extracellular matrix. This is followed by the inflammatory phase, during which immune cells such as neutrophils and macrophages migrate to the wound area to remove debris, pathogens, and damaged tissue while releasing cytokines and growth factors. The proliferative phase is characterized by fibroblast proliferation, collagen deposition, angiogenesis, and re-epithelialization, leading to the formation of granulation tissue. Finally, during the remodeling phase the newly formed tissue undergoes structural reorganization, with collagen fibers aligning and strengthening over time to restore mechanical stability [160]. A critical component of successful tissue regeneration is angiogenesis, the formation of new blood vessels from pre-existing vasculatures [161,162]. Adequate vascularization ensures delivery of the oxygen, nutrients, and signaling molecules necessary for cell survival and tissue growth. Hydrogel scaffolds can promote angiogenesis by providing a porous structure that supports endothelial cell migration and by serving as carriers for pro-angiogenic factors such as vascular endothelial growth factor (VEGF) and basic fibroblast growth factor (bFGF) [163]. Additionally, the tunable mechanical and biochemical properties of hydrogels facilitate tissue integration by encouraging interactions between the scaffold and surrounding host tissue, ultimately leading to gradual scaffold degradation and replacement by newly formed tissue [164]. Hydrogel design for 3D/4D printed scaffolds involves tuning parameters such as polymer composition, crosslinking density, degradation rate, and mechanical stiffness. These factors directly influence scaffold properties like porosity, swelling behavior, and shape-morphing ability. In turn, scaffold properties regulate biological processes; for instance, pore size and stiffness affect cell infiltration, vascularization, and immune cell recruitment. Angiogenesis is highly sensitive to scaffold architecture and biochemical cues; optimal pore interconnectivity and mechanical compliance can facilitate endothelial cell migration and capillary sprouting, while controlled release of angiogenic growth factors such as VEGF or FGF can further enhance vessel formation within the scaffold. In 4D-printed hydrogels, dynamic shape changes or stimuli-responsive behavior can further guide tissue regeneration by promoting directional cell growth, controlling local biochemical cues, or releasing immunomodulatory factors, creating a feedback loop between scaffold mechanics and biological activity. These properties make 3D and 4D hydrogel scaffolds particularly promising for the treatment of chronic wounds, including diabetic ulcers, burn injuries, and pressure sores [165]. Diabetic ulcers are characterized by impaired vascularization, neuropathy, and persistent inflammation, all of which significantly delay healing [166,167]. Hydrogel scaffolds can address these challenges by maintaining a moist wound environment, delivering therapeutic agents such as growth factors or antimicrobials, and supporting cellular activity essential for tissue regeneration [168,169,170]. In burn injuries, hydrogel scaffolds can function as protective dressings while simultaneously promoting re-epithelialization and dermal regeneration [171,172]. Similarly, in pressure sores, which are commonly associated with prolonged immobility, hydrogel-based systems can enhance tissue repair by improving local hydration, reducing infection risk, and stimulating angiogenesis and cell proliferation [173]. Overall, the integration of 3D and 4D printed hydrogel scaffolds into wound management strategies represents a promising advancement in regenerative medicine. By combining structural support, bioactive molecule delivery, and adaptive responsiveness to the wound microenvironment, these biomaterials provide innovative solutions for improving tissue healing and restoring function in both acute and chronic wounds.

### 4.3. Advanced Functionalization of Printed Hydrogels

Advanced functionalization strategies are increasingly used to enhance the biological performance of 3D/4D-printed hydrogel scaffolds for regenerative medicine [174]. One major area of development involves the incorporation of growth factors into printed hydrogel scaffolds to stimulate tissue regeneration [175]. Growth factors such as vascular endothelial growth factor (VEGF) or epidermal growth factor (EGF) can be embedded directly in hydrogel matrices or delivered through nanoparticle carriers to achieve sustained and localized release [176]. This controlled delivery enhances angiogenesis, cell proliferation, and tissue-specific differentiation, thereby improving the regenerative potential of engineered constructs. Nanoparticle-mediated delivery systems are particularly effective because they protect growth factors from degradation and allow for temporal control over release kinetics within the scaffold microenvironment. However, the bioactivity and stability of these growth factors remain a critical concern, as exposure to mechanical stress during printing or environmental factors post-implantation can reduce their efficacy. Furthermore, dose-dependent toxicity may arise if growth factor concentrations exceed physiological thresholds, potentially triggering aberrant cell proliferation or inflammatory responses.

Another important advancement is the development of stem cell-laden bioinks for cell-loaded bioprinting [177]. Hydrogels such as gelatin methacryloyl (GelMA), alginate, or hyaluronic acid derivatives can encapsulate stem cells, including mesenchymal stem cells (MSCs), endothelial progenitor cells, or induced pluripotent stem cells [178,179]. These constructs allow for spatial patterning of living cells during the printing process, promoting the formation of functional tissue-like structures. Studies have demonstrated that hydrogel-based scaffolds containing stem cells can enhance vascularization, accelerate wound healing, and facilitate integration with host tissue after implantation. Yet, stem cell-laden scaffolds introduce additional complexities, including variable cell viability during printing, immunogenic responses, and challenges in standardizing cell sources, which can compromise reproducibility and therapeutic outcomes.

Nanotechnology has also played a crucial role in the functionalization of printed hydrogels through the development of nanocomposite bioinks [180]. Incorporation of nanoparticles such as metal oxides, silica, or polymeric nanoparticles can improve the mechanical strength, printability, and bioactivity of hydrogel scaffolds [181]. Additionally, nanoparticles can act as carriers for therapeutic molecules, enabling targeted delivery of growth factors, genes, or drugs [182]. Nevertheless, potential cytotoxicity and long-term biocompatibility of nanoparticles remain concerns. Dose-dependent cellular stress, oxidative damage, or unintended immune activation can arise depending on nanoparticle type, size, and surface chemistry, necessitating careful preclinical evaluation.

The integration of bioactive molecules and peptides represents another promising strategy for hydrogel functionalization. Short bioactive peptides such as RGD motifs or peptides derived from bone morphogenetic proteins (BMPs) can be incorporated into hydrogel networks or grafted onto scaffold surfaces to promote cell adhesion and lineage-specific differentiation [183]. These molecules can also stimulate mineralization and osteogenic activity in bone tissue engineering applications. However, the stability and controlled presentation of these bioactive cues remain challenging, as enzymatic degradation and diffusion can diminish their efficacy over time.

The controlled presentation of biochemical signals within printed hydrogels enables the design of biomimetic scaffolds that closely replicate the signaling environment of native tissues. Overall, the integration of growth factors, stem cells, nanoparticles, and bioactive peptides into 3D/4D printed hydrogels has significantly expanded the functionality of these scaffolds. These advanced multifunctional constructs provide controlled biochemical cues, enhanced mechanical properties, and improved cellular responses, positioning them as progressive platforms for next-generation regenerative medicine and personalized tissue engineering therapies.

Despite these advances, the clinical translation of advanced hydrogel scaffolds remains limited by significant challenges [184]. Manufacturing complex 3D/4D printed or cell-laden constructs at a clinically relevant scale while maintaining consistent mechanical properties, bioactivity, and structural fidelity is still difficult, particularly when sensitive components such as stem cells or growth factors are involved. Reproducibility is further complicated by variability in bioink composition, printing parameters, and cell sources, which can lead to inconsistent biological outcomes [185]. Regulatory approval presents another substantial barrier, as multifunctional scaffolds often combine biomaterials, living cells, and biologically active molecules, placing them under stringent combination-product regulations. High production costs limited long-term clinical data, and potential risks such as dose-dependent toxicity, immune reactions, and loss of bioactive molecule stability further constrain clinical adoption.

To sum up, while the integration of growth factors, stem cells, nanoparticles, and bioactive peptides into 3D/4D printed hydrogels significantly enhances scaffold functionality for targeted drug delivery and wound healing, a critical evaluation of safety, stability, reproducibility, and regulatory pathways is essential. Addressing these limitations through rigorous preclinical testing, standardized manufacturing protocols, and careful dose optimization will be essential to translating these multifunctional hydrogel systems into safe and effective regenerative therapies.

## 5. Applications of Hydrogel Scaffolds

### 5.1. Skin Tissue Engineering

Chronic wounds are wounds that fail to complete normal healing, remaining in an inflammatory state with elevated bacteria, cytokines, reactive oxygen species, and proteases [186]. Common types include pressure sores, venous ulcers, and diabetic foot wounds, often affecting patients with diabetes, obesity, or limited mobility. Traditional dressings such as gauze and bandages have limitations in moisture control, bioactivity, mechanical strength, and sustained therapeutic delivery. Advanced dressings, including scaffolds, films, nanofiber meshes, and hydrogels, can offer improved outcomes, with scaffolds being particularly effective at mimicking the extracellular matrix, supporting cell adhesion, proliferation, and differentiation processes, and enabling controlled degradation and release of bioactive agents. Ideal scaffolds are biocompatible, biodegradable, non-immunogenic, and mechanically suitable for tissue repair [187].

Tissue engineering is a promising regenerative strategy aimed at repairing damaged tissues, employing both natural and synthetic biomaterials to support tissue or organ growth ex vivo [188]. Among various 3D scaffolds, hydrogel-based scaffolds and constructs are particularly valuable for skin tissue regeneration due to their high moisture retention, porosity, biocompatibility, biodegradability, and biomimetic properties. Moreover, bioactive polymer hydrogels can be functionalized with drugs, nanoparticles, or stem cells to enhance their therapeutic potential [189].

Natural bioactive compounds with antimicrobial, antioxidant, and anti-inflammatory properties are increasingly being explored for numerous therapeutic applications, including the fabrication of 3D printed scaffolds as customizable hard and soft tissue substitutes. Strategic integration of bioactive compounds into 3D/4D printed hydrogel scaffolds is a beneficial approach that can modulate biological responses and enhance tissue regeneration while also conferring antioxidant and antibacterial functions, resulting in improved wound healing outcomes. One concrete application utilized para-coumaric acid (P-CA), a plant-derived phenolic compound that exhibits potent antioxidant activity that attenuates oxidative stress, a key barrier to wound healing. A biocompatible scaffold for wound repair was developed by incorporating P-CA into a chitosan-based matrix using hot melt extrusion and fused deposition modeling 3D printing, with chitosan (CS), polyethylene oxide (PEO), and cryo-milled polycaprolactone (PCL) utilized to optimize printability and mechanical strength. The PCL/CS/P-CA/PEO scaffold showed high dimensional accuracy and mechanical integrity, sustained P-CA release over three days, and synergistic antibacterial activity against *E. coli*. These findings highlight the potential of P-CA-loaded CS multifunctional scaffolds for enhanced wound healing [190].

Hydrogel-based tissue engineering in combination with exogenous stem cells offers considerable potential for enhancing wound regeneration. Nevertheless, conventional tissue engineering materials often fail to provide an optimal microenvironment. To address this, enzymatically cross-linked hyaluronic acid hydrogels have been designed to support acute wound healing. In one such approach, dopamine was grafted onto hyaluronic acid followed by enzymatic cross-linking using horseradish peroxidase and hydrogen peroxide, yielding a porous and loosely structured hydrogel. This material exhibited adhesive and self-healing properties, creating a microenvironment highly compatible with exogenous bone marrow-derived mesenchymal stem cells. In vivo experiments demonstrated that the hydrogel scaffolds markedly accelerated the repair of full-thickness cutaneous wounds to promote appendage regeneration, including hair follicles, while minimizing scar formation [191].

**Figure 7:**
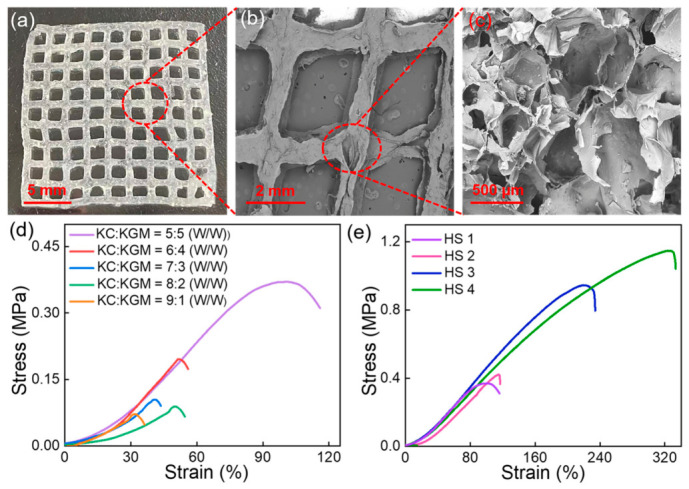
(**a**) Photograph of the FLA@ZIF-8/KC@KGM hydrogel scaffold. (**b**,**c**) SEM images showing scaffold microstructure. (**d**) Tensile strain curves for scaffolds with varying KC/KGM ratios. (**e**) Tensile strain curves for scaffolds with different FLA@ZIF-8 nanoparticle loadings. Reprinted from [192].

**Figure 8:**
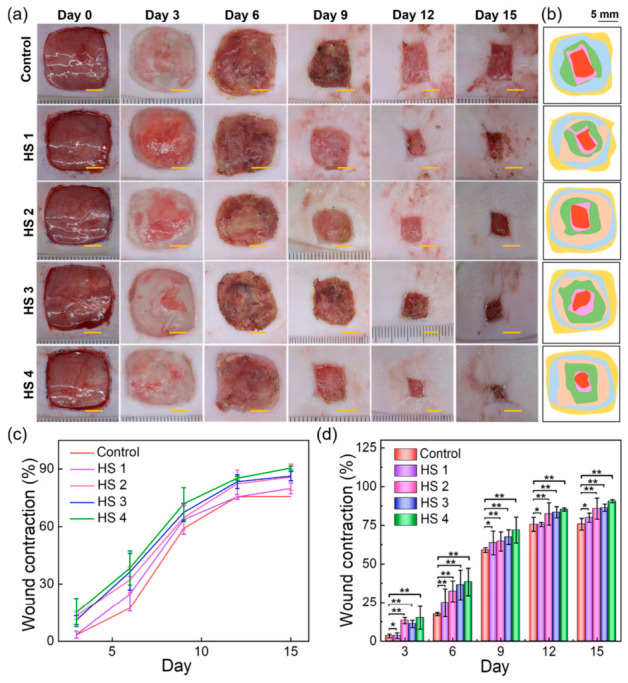
(**a**) Photographs showing the wound healing progression (scale bar: 5 mm). (**b**) Simulated wound closure traces and contraction at days 0, 3, 6, 9, 12, and 15 for control and HS 1–4 groups (scale bar: 5 mm). (**c**,**d**) Wound contraction rates over time for all groups; data are mean ± SD (*n* = 5), * *p* < 0.05, ** *p* < 0.01. Reprinted from [192].

Bacterial-infected skin wounds remain a major clinical challenge, highlighting the need for dressings with strong antibacterial and regenerative properties. 3D-printed hydrogel scaffolds offer precise control over architecture and porosity, enabling tailored cell infiltration and nutrient transport to support tissue regeneration. Incorporating bioactive compounds or growth factors into these scaffolds can provide sustained antimicrobial activity while promoting angiogenesis and wound closure. The tunable mechanical properties of hydrogels also allow them to conform to irregular wound sites, maintaining a moist environment that accelerates healing. In this regard, a multifunctional 3D-printed hydrogel scaffold was developed using a κ-carrageenan (KC)/konjac glucomannan (KGM) matrix loaded with flavanone@ZIF-8 nanoparticles (FLA@ZIF-8 NPs) (Figure 7) [192]. The dual-network structure enhances mechanical stability and adaptability, while controlled release of Zn^2+^ and flavanone under mildly acidic conditions provides sustained antibacterial activity and promotes cell proliferation. In vivo studies in a rat infected wound model demonstrated that the scaffold significantly accelerated healing, underscoring its potential for treating bacterial-infected wound (Figure 8).

While the scaffold showed promising short-term efficacy, its long-term in vivo performance, biodegradation, and systemic safety remain unresolved and require further investigation. For clinical translation, understanding the kinetics of biodegradation, long-term biocompatibility, and potential chronic inflammatory responses is essential. Short-term success in rat models does not guarantee safety or efficacy in humans.

For rapid wound healing, new multifunctional 3D-printable hydrogels have been developed based on Zn/tannic acid-reinforced glycol-functionalized chitosan. Double cross-linking was use to enhance mechanical strength, adhesion, and viscoelasticity while providing strong antibacterial activity against *B. subtilis* and *E. coli* and excellent biocompatibility with human dermal fibroblasts and macrophages. The hydrogels promoted M2 macrophage polarization, accelerated collagen deposition, and enhanced re-epithelialization in rats, demonstrating their potential for rapid regeneration of bacterially infected wounds [193].

**Figure 9:**
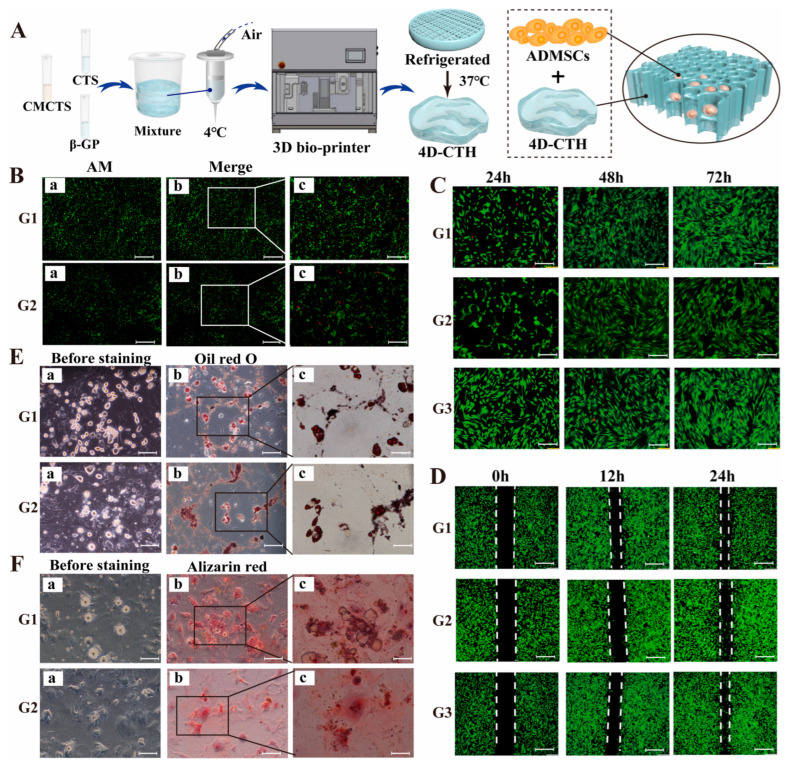
Cytocompatibility evaluation of 4D-CTH. (**A**) Schematic of ADMSCs cultured on 4D-CTH. (**B**) Live/dead staining (AM/PI) of ADMSCs on 4D-CTH and CTH (scale bar: 500 μm). (**C**) AM/PI images after 24, 48, and 72 h co-incubation (scale bar: 200 μm). (**D**) Wound-healing assay images at 12 h and 24 h (scale bar: 500 μm). (**E**) Oil Red O staining showing adipogenic differentiation of ADMSCs; scale bars: 100 μm (left, middle) and 50 μm (right). (**F**) Alizarin Red staining showing osteogenic differentiation of ADMSCs (images (**Ea**,**Fa**) were taken before staining); scale bars: 100 μm (**left**,**middle**) and 50 μm (**right**). Reprinted from [194].

Diabetic wounds are hindered by impaired stem cell function and poor vascularization, limiting the effectiveness of traditional therapies; 4D-printed hydrogel scaffolds offer a dynamic platform that can modulate the local microenvironment by delivering targeted agents to enhance stem cell survival, migration, and differentiation. This integration of advanced biomaterials and molecular therapy provides a promising strategy to restore regenerative capacity and promote angiogenesis in chronic diabetic wounds. A recent study found that piRNA-hsa-32182, which is highly expressed in diabetic wounds, inhibits adipose-derived mesenchymal stem cell (ADMSC) migration and differentiation [194]. Using a 4D-printed hydrogel combined with a piRNA-hsa-32182 antagomir, researchers created a supportive microenvironment that enhanced ADMSC survival, migration, collagen deposition, and angiogenesis, offering a promising new approach for diabetic wound healing (Figure 9).

**Figure 10:**
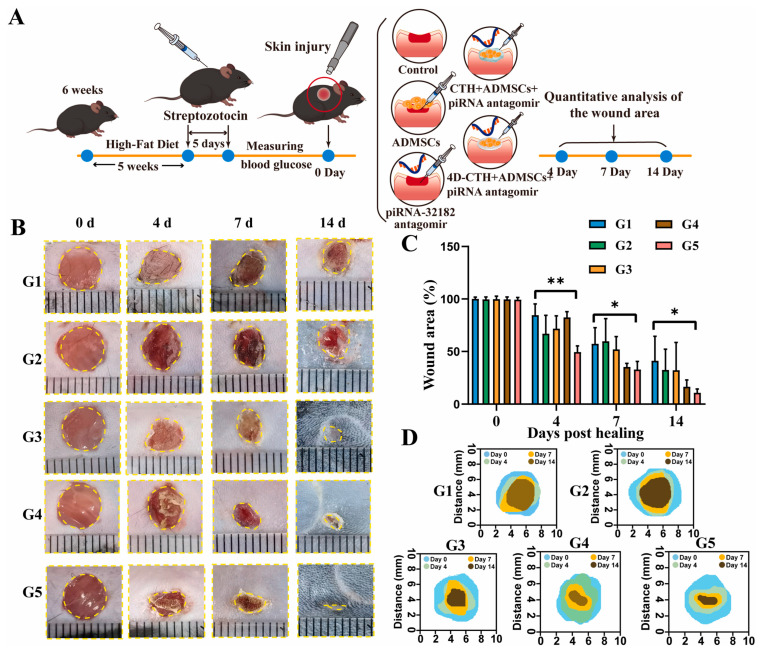
Effects of various therapies on in vivo diabetic wound healing. (**A**) Schematic of the animal experiment. (**B**) Representative wound images at days 0, 4, 7, and 14 under different treatments. (**C**) Quantitative analysis of wound area. (**D**) Comparison of wound healing rates among groups. Data are mean ± SD (n = 4); * *p* < 0.05, ** *p* < 0.01 vs. control (ANOVA). Reprinted from [194].

The results of in vivo tests performed on groups of mice showed that after 14 days of treatment, the success of piRNA antagomir therapy combined with 4D-CTH loaded with ADMSCs was confirmed in promoting diabetic wound healing, restoring the mechanical properties of the skin, and nerve regeneration (Figure 10).

While the above study demonstrates an innovative approach to 4D-printed stem cell molecular therapy for diabetic wounds, long-term integration, sustained stem cell function, scaffold biodegradation, and chronic safety (repeated or long-term use) under high glucose conditions remain unresolved and require further investigation. Diabetic wounds are chronic and recurrent, unlike acute wounds in animal models; therefore, ensuring durable scaffold function, stem cell survival, and efficacy of the molecular intervention over long periods of time is crucial for clinical translation.

Table 3 concentrates the main works reviewed in this subsection and highlights advances such as antibacterial activity, stem cell support and 3D/4D bioprinting that improve wound healing and tissue regeneration.

### 5.2. Minimally Invasive Implantation

The introduction of customized hydrogel scaffolds with complex structures has shown promise in various biomedical engineering applications, including medical devices, drug delivery, and biological engineering. However, the introduction of bulky 3D scaffolds can cause local injuries [195]. With the advancement of minimally invasive surgical techniques, the development of scaffolds that can be delivered in a compact form and then autonomously expand to fit the implantation site would be extremely beneficial for intraoperative management as well as for reducing postoperative pain and speeding recovery [196]. State-of-the-art biomaterials developed as smart hydrogel inks allow for 4D printing of scaffolds capable of programmable deformations and that are responsive to stimuli, being able to change their shape in response to environmental cues and dynamically adapt to biological conditions [197,198]. This capability holds significant promise for regenerative medicine, as it allows implants and tissue engineering constructs to better and more easily integrate with the body and support tissue regeneration [199].

Minimally invasive implantation typically uses two strategies: compressing a 3D-printed scaffold into a 2D sheet that later expands in the body once at the target site, or implanting a 2D sheet that folds into 3D in situ in response to various stimuli. However, limited 2D-to-3D deformation still causes trauma, which could be further reduced by using 1D-shaped scaffolds to facilitate insertion via a transcatheter.

**Figure 11:**
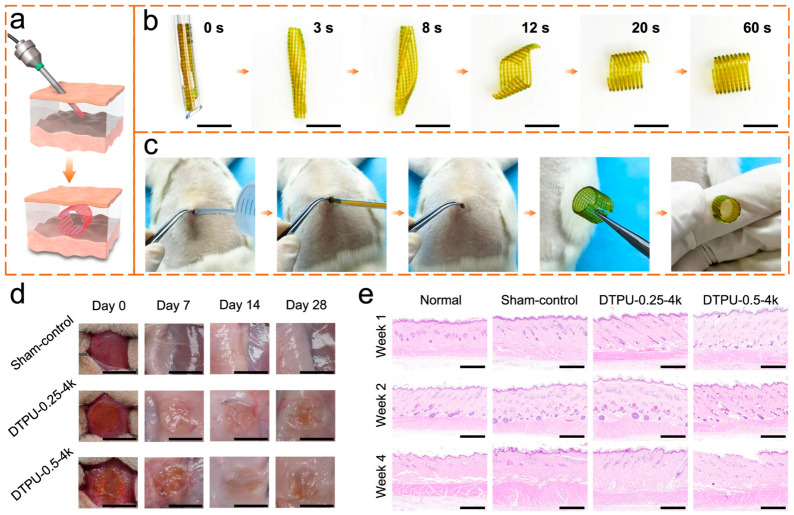
Images showing 4D-printed scaffolds for transcatheter delivery and multidimensional subcutaneous deformation. (**a**) Schematic. (**b**) Digital images of delivery and multi-stimuli responsiveness. (**c**) Subcutaneous 1D-to-3D transformation in SD rats. (**d**) Macroscopic views of implants and surrounding tissues over time. (**e**) Histology of DTPU-0.25-4k and DTPU-0.5-4k scaffolds at different time points. Scale bars: (**b**,**d**) 10 mm, (**e**) 1 mm. Reprinted from [200].

In one study, 4D-printed dynamic thermoset polyurethanes were designed as body temperature-triggered shape-memory scaffolds with programmable water-induced 3D transformation [200]. The 2D printed scaffold can be fixed into a 1D shape for minimally invasive insertion, recover to 2D at body temperature, then swell into the target 3D structure, with a soft-to-rigid transition enhancing structural support. Successful minimally invasive implantation of the DTPU printed scaffolds was demonstrated through in vitro and in vivo experiments in rats (Figure 11) [200].

The DTPU 4D printed scaffolds fabricated and investigated in this study demonstrated innovative multi-stimuli responsive properties for minimally invasive tissue support. However, long-term in vivo mechanical integrity, shape retention, and tissue integration remain unresolved and require further investigation prior to clinical application. Minimally invasive scaffolds are intended for clinical translation, which requires consistent long-term functionality and safe integration into human tissue. Short-term demonstrations of shape recovery and swelling–stiffening behavior are promising, but clinical applicability remains uncertain without a full understanding of lastingness and host response.

Table 4 presents recent advances in the field of 3D/4D printed hydrogel scaffolds for minimally invasive implantation, with an emphasis on in situ fabrication approaches that enable compact delivery and subsequent transformation in the body. These approaches can improve the adaptability, precision, and clinical applicability of scaffolds in regenerative medicine.

### 5.3. Bone Regenerative Medicine

Despite the fact that 3D printing is increasingly used for fabricating scaffolds in bone tissue engineering, creating hydrogel-based bioinks that possess both bioactivity and self-healing capabilities still presents significant challenges. Attempts have been made to fabricate 3D bone scaffolds based on osteogenic composite hydrogel ink, which was shown to maintain its self-healing properties after incorporation of bioactive glass particles [201]. Bone diseases frequently exceed the body’s natural healing capacity, highlighting the need for improved scaffolds in bone tissue engineering. A highly porous (>70%) biomimetic scaffold was fabricated based on a pH-sensitive hydrogel containing curcumin-loaded hydroxyapatite. The system exhibited pH-dependent biphasic drug release that was minimal at physiological pH and rapid under acidic conditions while maintaining good mechanical strength and biocompatibility (cell viability > 80%), showing promise for bone repair and controlled drug delivery [202]. Bioactivity evaluation in SBF solution indicated the formation of HA particles on the composite hydrogels. This new formulation showed promising potential in obtaining extrusion-based 3D printing hydrogel inks for bone constructs.

**Figure 12:**
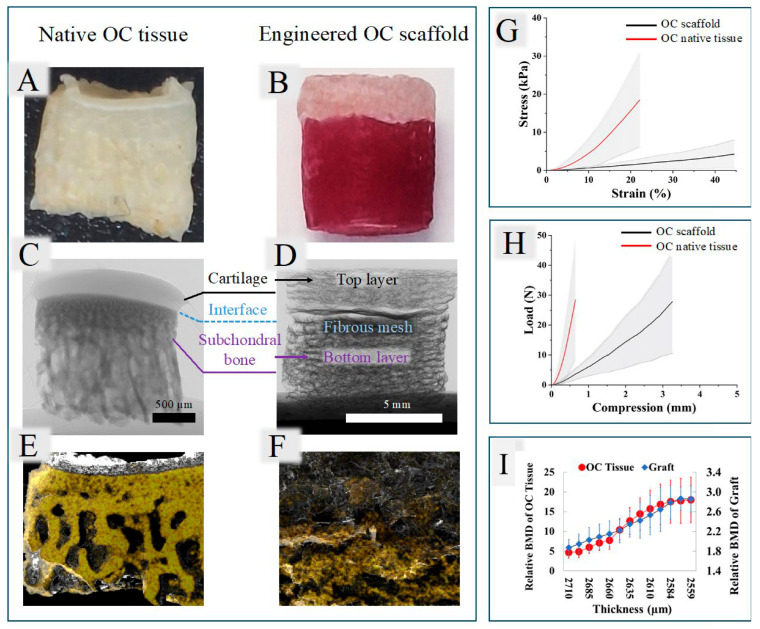
Mineral distribution in native OC tissue and scaffolds: optical images (**A**,**B**), micro-CT reconstructions (**C**,**D**), and EDX elemental maps (**E**,**F**). Mechanical analysis includes stress–strain (**G**) and compression (**H**) curves with interface mineral gradient comparison (**I**). Shaded areas indicate standard deviation. Reprinted from [203].

Osteochondral (OC) tissue consists of cartilage and the underlying subchondral bone and is essential for proper knee joint function. Damage to OC, often caused by trauma, aging, or diseases such as osteoarthritis, impairs mobility and causes chronic pain. Because articular cartilage lacks blood vessels, its natural ability to heal is extremely limited, making OC damage largely irreversible. Graded osteochondral scaffolds have been fabricated via sequential 3D printing to mimic the bone–cartilage interface [203]. The bottom layer (gelatin/oxidized alginate with hydroxyapatite) promotes bone formation, while the top layer with smaller pores supports cartilage regeneration. An electrospun PCL membrane is inserted between layers to mimic the tidemark and prevent cell migration. The scaffolds showed good cell viability and mechanical properties comparable to native OC tissue. Rabbit knee OC tissue was used to validate the fabricated scaffold (Figure 12) [203].

**Figure 13:**
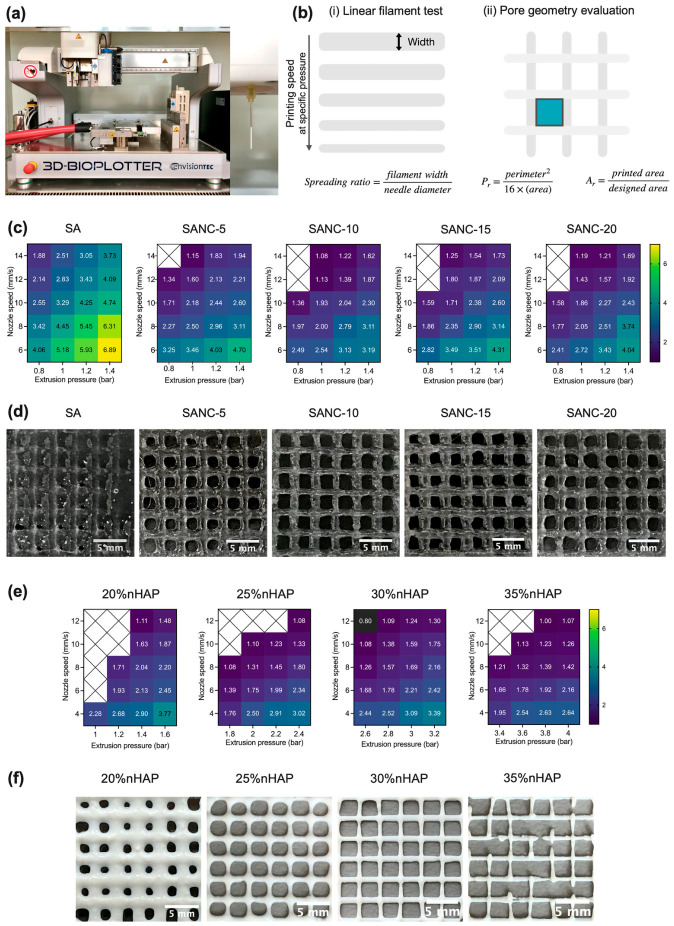
Printability and shape fidelity of SANC and HSANC hydrogels. (**a**) The 3D printing setup using a 0.6 mm blunt needle. (**b**) Linear filament spreading and pore geometry evaluation for two-layer cross-hatched grids (printability index, Pr; pore area accuracy, Ar). (**c**,**e**) Filament spreading ratio under different printing conditions. (**d**,**f**) Pore geometry of planar structures printed under optimal conditions for SANC and HSANC-10 hydrogels. Reprinted from [204].

**Figure 14:**
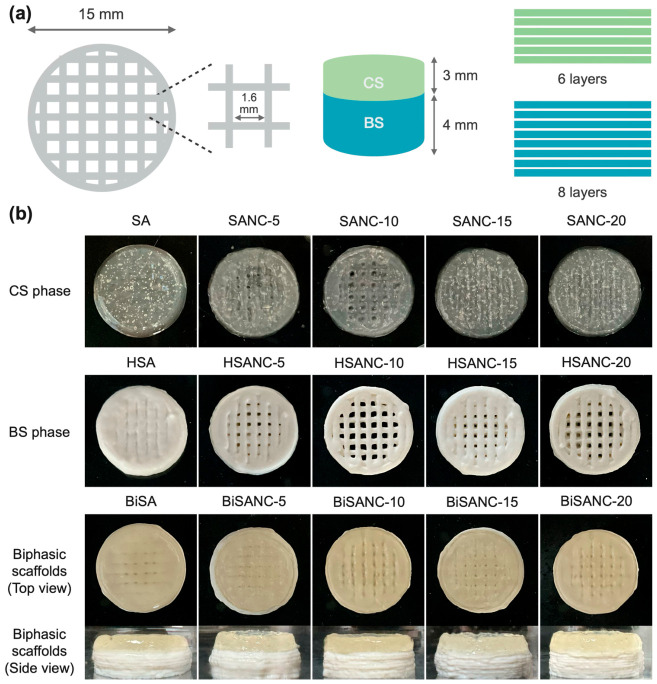
Hierarchical structure of the 3D-printed scaffolds. (**a**) Diagram showing the design strategy used for fabricating 3D scaffolds. (**b**) Images of the CS phase, BS phase, and the biphasic scaffold, presented from both top and side perspectives. Reprinted from [204].

Nanomaterials used as reinforcement are essential for improving the strength, printability, and overall structural stability of hydrogels used in scaffold fabrication, including for the environmentally friendly fabrication of scaffolds for regenerative medicine applications. TEMPO-oxidized nanocellulose (TONC) obtained from coffee parchment was added to sodium alginate hydrogels as a reinforcing component for extrusion-based 3D printing of biphasic osteochondral scaffolds [204]. Following fabrication, the TONC-enhanced printed scaffolds demonstrated improved geometric accuracy and shape stability along with notable functional performance. These scaffolds showed increased water absorption, diffusion-regulated drug release, a higher Young’s modulus, and strong biocompatibility that promoted cell growth and proliferation. Overall, coffee parchment-derived nanocellulose functioned as a sustainable reinforcement for 3D printed scaffolds, improving printability and extrusion behavior while improving scaffold stability, mechanical properties, and ability to achieve controlled drug delivery. The fabrication of biphasic scaffolds obtained with a 4D printer and the evaluation of the printability, structural accuracy of the printed constructs, including the formation of linear filaments and pore geometry for the sodium alginate (SA)/nanocellulose (NC)-based hydrogel inks, SANC (with different SA concentrations ranging from 0–20%), are shown in Figure 13a–d. For the evaluation of the HSANC hydrogels made for the bone-like layer, the optimal concentration of nanohydroxyapatite (nHAP) (ranging from 20–35%) was identified and its influence on the geometry of the resulting scaffold was analyzed (Figure 13e,f) [204]. Figure 14 also shows biphasic scaffolds (BiSANC 5–20) that were 3D printed in a cylindrical shape, resembling bone at the base (BS) using HSANC hydrogels and SANC hydrogels for the cartilage-like upper phase. The study represents a significant advancement, yet certain questions and uncertainties still persist. The most pressing unresolved feature is the lack of data on long-term scaffold behavior under physiological conditions, including hydration-driven mechanical changes, degradation, and structural stability. This is crucial for clinical translation in osteochondral tissue engineering, where the scaffold must maintain both mechanical support and biofunctionality over weeks to months while interacting with surrounding tissues.

**Figure 15:**
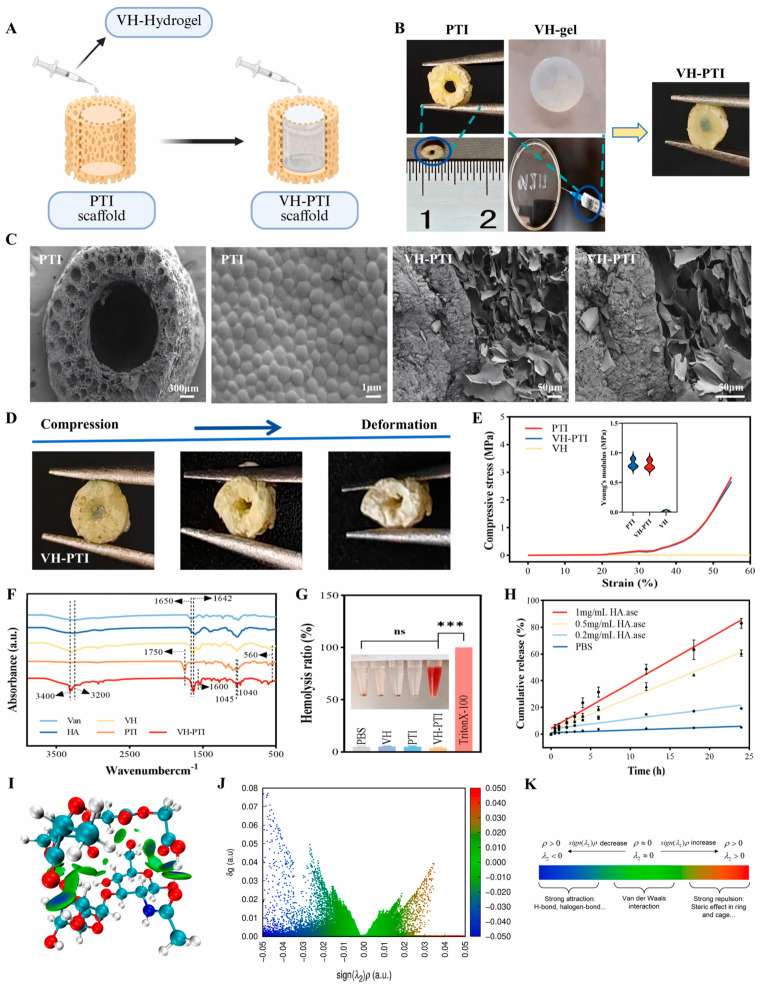
Fabrication and characterization of VH-PTI composite scaffolds. (**A**) Schematic of VH-PTI scaffold assembly. (**B**) Scaffold appearance and composition. (**C**) SEM images of PTI and VH-PTI scaffolds. (**D**) Compression deformation of VH-PTI scaffold. (**E**) Stress–strain curves and Young’s modulus. (**F**) FTIR spectra of related components and scaffolds. (**G**) Hemolysis test. (**H**) Van cumulative release profiles. (**I**–**K**) Interaction analysis of HA-PLGA, including δginter map, Sign (λ_2_)ρ IGMH scatter plot, and interaction color scale. Data are mean ± SD (n = 3, *** *p* < 0.001). Reprinted from [206]. ns = not significant.

Artificial implants are the most effective method for repairing bone fractures, especially in orthopedics and dentistry. Current research focuses on creating multifunctional implants that mimic natural bone tissue; however, designing such “smart” implants is challenging because it is not fully understood how material properties influence cell behavior and bone regeneration [205]. For the intelligent prevention and treatment of bone implant infection and promotion of bone repair, architectures inspired by nature, such as the structure of the beehive, have been designed [206]. An adaptive honeycomb-mimetic hyaluronic acid hydrogel composite scaffold was developed as an intelligent enzyme-responsive unit. The scaffold (VH-PTI) demonstrated dynamic antibacterial activity, promoted stem cell growth and differentiation, and successfully treated MRSA-infected rabbit tibial defects while supporting bone regeneration. The scaffold features a 3D-printed PTI outer layer for structural support, while its core contains vancomycin-loaded HA hydrogel that responds to bacterial hyaluronidase (Figure 15) [206]. This allows for on-demand antibiotic release at infection sites and presents minimal degradation when no infection is present, ensuring targeted post-implantation infection control.

This study reveals a highly innovative VH-PTI composite scaffold that addresses two major clinical challenges (bacterial infection and delayed bone regeneration) through a smart combination of structural design and enzyme-sensitive drug delivery, achieving dual functionality that mimics natural defense and repair mechanisms. However, expanding the evaluation to more bacterial strains, infection durations, and more clinically relevant large animal models is essential to fully validate the translational potential of the scaffold.

**Figure 16:**
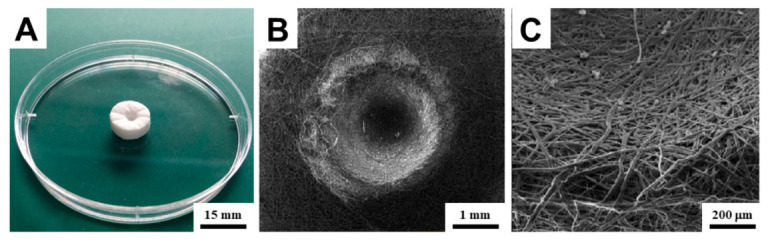
A 3D scaffold in the form of disc containing a pressed trap hole. (**A**) Real image of the scaffold showing the pressed hole. (**B**) SEM image of the pressed hole structure. (**C**) Higher-magnification SEM image illustrating the detailed morphology of the hole wall. Reprinted from [208].

Another promising strategy involves the combination of 3D biodegradable scaffolds with injection-based bioprinting of hydrogels [207]. This approach may generate a synergistic effect by integrating the favorable mechanical strength and structural stability of fibrous polycaprolactone scaffolds with the precise delivery of a highly hydrophilic hydrogel cell suspension. Such a system can enhance cell proliferation within the scaffold interior and support the formation of more compact tissue structures [208]. Initially, an optimized 3D porous scaffold was fabricated from a composite of melt-blown microfibers and electrospun nanofibers, providing a structure suitable for effective cell adhesion (Figure 16). In the subsequent step, the scaffolds were injection-bioprinted with a hyaluronan-based hydrogel cell suspension, which filled the fibrous network and established a favorable hydrophilic environment. This combination demonstrated high biocompatibility and resulted in an increased number of viable cells immediately after seeding, indicating improved cell proliferation and more rapid formation of confluent cell layers compared with conventionally seeded scaffolds. The results suggest that collagen-containing hyaluronan hydrogel is adequate for bioprinting human osteoblasts within the studied scaffold, and that this combined scaffold–bioprinting approach shows promise for bone regenerative medicine.

**Figure 17:**
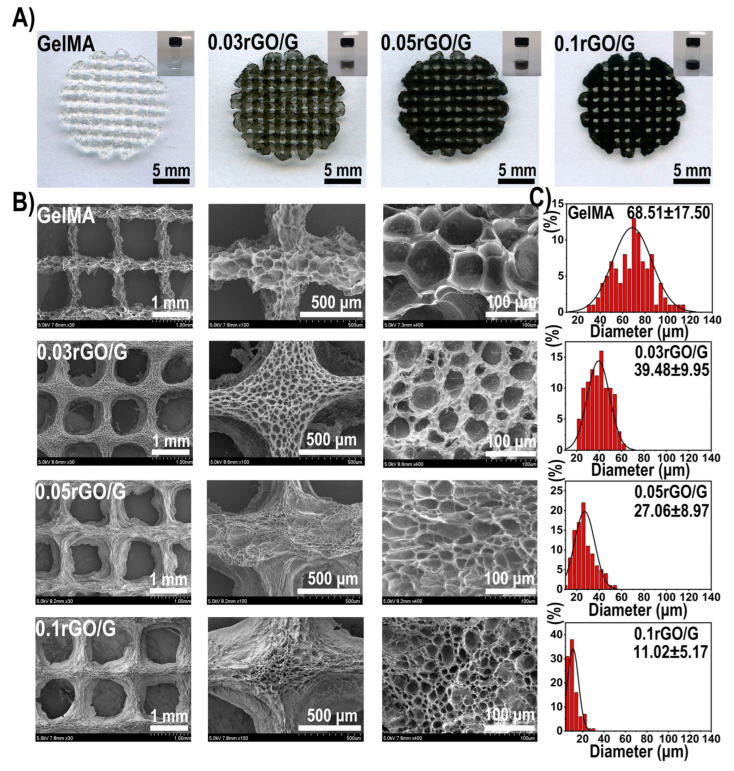
(**A**) Images of GelMA precursor solutions and 3D-printed scaffolds with varying rGO concentrations. (**B**) SEM image of the rGO composite hydrogel scaffold. (**C**) Quantitative analysis of hydrogel pore size. Reprinted from [209].

A hydrogel scaffold such as 3D printed rGO/GelMA-based bioink co-loaded with Schwann cells and bone marrow-derived mesenchymal stem cells (BMSCs) promotes simultaneous nerve and bone regeneration (Figure 17) [209]. rGO enhances osteogenic and neurogenic differentiation, mechanical strength, and cell adhesion, demonstrating high cell survival, low inflammation, and strong expression of bone and neuronal markers, which offers a promising strategy for multicellular delivery, functional tissue regeneration, and functionalized bone repair. Macroscopic images of GelMA precursor solutions and 3D scaffolds with different rGO concentrations are shown in Figure 17 together with the morphology of the lyophilized scaffolds.

A comparative overview of advanced 3D/4D-printed hydrogel scaffolds for bone and osteochondral tissue engineering is provided in Table 5, highlighting material composition, fabrication strategies, functional properties, mechanical performance, drug or bioactive delivery capabilities, and biological outcomes.

### 5.4. Dental Applications

Periodontal disease is a prevalent global oral health issue, affecting 20–50% of the population. Its pathogenesis involves the host immune response to periodontopathogenic bacteria, leading to inflammation, alveolar bone loss, and progressive periodontal attachment loss, which can result in tooth loss [210,211]. Treatment can be initially non-surgical (scaling and root planing combined with oral hygiene review) or later through resective or regenerative surgery. Given the limitations of current therapies, innovative approaches are needed [212].

Tissue engineering holds significant promise for the regeneration of oral tissues, including periodontal and alveolar bone structures, with scaffolds serving an important role by supporting the attachment and proliferation of oral stem cells and osteoprogenitor cells [213]. Various scaffold fabrication methods have been explored, including gas foaming, freeze-drying, and fiber bonding, yet 3D printing has emerged as the most effective approach, offering precise control over scaffold architecture to promote uniform tissue development [214]. Biomaterials must be biocompatible and integrate seamlessly with the oral environment to restore functional healthy tissue without scarring [215]. Scaffolds can be utilized at multiple stages of oral therapy, including for reducing inflammation, combating microbial biofilms, or promoting tissue regeneration. Despite these advances, challenges persist: some 3D-printed scaffolds exhibit brittleness, insufficient mechanical strength, or limited biocompatibility, which can restrict their effectiveness in oral tissue restoration. Through an innovative approach to the fabrication process, 3D printed chitosan-based scaffolds with *Scutellariae baicalensis* extract were prepared for the personalized treatment of periodontal diseases. The extract was rich in flavones and increased the effectiveness of the treatment due to its antibacterial, antifungal, antioxidant and anti-inflammatory properties [216]. The test results indicated the possibility of developing these scaffolds as a personalized dressing with anti-periodontal effect.

**Figure 18:**
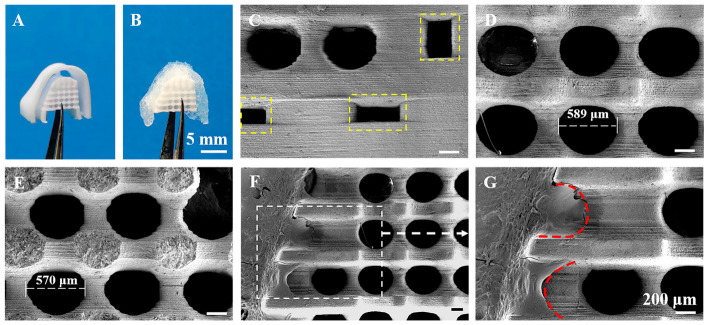
Morphology and structural characterization of biphasic hierarchical architectures. (**A**,**B**) Outward appearance before and after integration with the hydrogel membrane. (**C**) SEM image of perforations at the connection area between the hydrogel mold and porous nCSi scaffold (yellow dotted box). (**D**,**E**) SEM images of outer and fracture surfaces. (**F**) Low-magnification view of the interface between the hydrogel membrane and bioceramic scaffold. (**G**) Enlarged interface region (white dotted box and arrow), with red dotted lines indicating hydrogel infiltration into the scaffold pores. Scale bar: 5 mm (**A**,**B**), 200 μm (**C**–**G**). Reprinted from [217].

Another very interesting study advanced a modularized hierarchical architecture with a hydrogel membrane bioceramic scaffold as a beneficial application for periodontal tissue regeneration in dogs [217]. A novel biphasic hierarchical scaffold was created using non-stoichiometric wollastonite (nCSi) and a silanized GelMA/Si-HPMC hydrogel membrane fabricated via digital light processing (DLP) and photocrosslinked hydrogel injection (Figure 18). The composite hydrogel showed favorable physico-chemical properties, with the membrane providing strong in vivo occlusion. In dog models of intraosseous periodontal defects, the hierarchical scaffold promoted significant periodontal regeneration. These findings highlight its potential as a next-generation oral implant and a precise guided tissue regeneration strategy for improving periodontal repair while reducing surgical complexity.

In one study, 3D-printed bilayer nanocomposite membranes formulated as nanocomposite hydrogels combining grape seed extract, simvastatin, and chitin nanocrystals showed strong mechanical properties, sustained dual drug release, cytocompatibility, and antibacterial activity. In vitro and in vivo studies confirmed the ability to promote bone regeneration and reduce inflammation, highlighting their potential for advanced periodontal tissue regeneration and infection control [218].

One of the main causes of tooth loss in periodontitis is the destruction of periodontal bone. Thus, by introducing new guided strategies, biomaterials can be obtained to direct the behavior of endogenous stem cells towards periodontal regeneration [219]. The design of a new dual-functional biomaterial targeting bone led to a hydrogel functionalized with osteogenic growth peptide-OGP and -YLL3 motifs (OGP-HAMA-YLL3) for the spatiotemporal control of stem cell orientation and osteogenic differentiation. The results of a further clinical study showed that the OGP-HAMA-YLL3 hydrogel scaffold was able to promote bone regeneration, reduce inflammation, and safely support periodontal repair mediated by endogenous stem cells, demonstrating strong translational potential. The system demonstrated strong preclinical efficacy in small (rat) and large (minipig) animal models, showing improved bone volume, mineralization, and reduced expression of inflammatory cytokines. However, the precise quantitative contribution of each component (HA matrix, OGP, YLL3, MSC recruitment) to the observed immunomodulatory and regenerative outcomes remains unclear. Furthermore, potential degradation products and effects on the periodontal microbiota over long periods of time have not been fully explored over the long term. Extensive in vivo follow-up would strengthen the translational potential of this approach.

### 5.5. Cancer Therapy

Melanoma, a very serious and aggressive skin cancer, is primarily treated by surgery, often combined with chemotherapy or radiotherapy due to relapse risks and tissue defects [220,221]. Conventional chemotherapies such as doxorubicin can cause severe side effects, and achieving high local drug concentrations remains challenging. Therefore, local, targeted drug delivery systems (DDS) that prevent tumor recurrence and support tissue regeneration are highly desirable. Creating scaffolds that simultaneously facilitate tissue regeneration and localized melanoma therapy via 3D-printed hydrogels and structures can allow for controlled and sustained drug release and modulation of the surgical microenvironment while also providing precise control over porosity, strut design, and layered structures. Novel 3D printed hydrogel blend scaffolds loaded with doxorubicin (DOX) as a model anticancer drug were investigated in [222]. Experiments with DOX-loaded rat bone marrow mesenchymal stem cell (rBMSC) and rat bone marrow mesenchymal stem cell (rBMSC) constructs aimed to inhibit postoperative melanoma recurrence and provide structural support for tissue regeneration. The results implied therapeutic promise; however, this needs to be followed by biological validation in future studies using an in vivo melanoma recurrence model, comparison with standard postsurgical treatments, and quantification of tumor cell killing versus healthy cell survival.

Colorectal cancer (CRC) remains a major global health burden with high incidence and mortality rates, particularly in advanced stages where the 5-year survival rate drops dramatically after metastasis appearance [223,224]. Although surgery is effective in early stages, current therapeutic strategies remain limited and the success rate of new anticancer drugs is still very low [225]. This is largely due to the poor predictive capacity of conventional preclinical models, which fail to replicate the complexity and heterogeneity of the tumor microenvironment (TME). Tumor progression is strongly influenced by dynamic interactions between cancer cells and the surrounding extracellular matrix (ECM), stromal, and immune components, which regulate processes such as invasion, metastasis, and drug resistance [226]. Therefore, there is a critical need for advanced studies focused on the development of more biomimetic platforms. In this context, hydrogel-based scaffolds have emerged as promising tools that can better mimic the structural and functional properties of the TME, enabling improved investigation of CRC progression and the development of more effective therapeutic strategies. On the other hand, the increasing demands of regenerative medicine have driven the development of new studies on new multifunctional biomaterials, leading to the emergence of advanced hydrogel systems designed to address complex clinical challenges. These innovative platforms must integrate multiple therapeutic functions such as tissue regeneration, immunomodulation, and anticancer activity into a single and coherent system. Hydrogels mimicking the ECM are essential for 3D cell culture. One study presented a functional 3D hydrogel construct for colon cancer cells designed to support physiological attachment, spreading and viability [227]. When comparing pure alginate and cross-linked alginate–gelatin (ADA-GEL) hydrogels, HCT116 colon cancer cells showed higher proliferation, network formation, and integrin-dependent adhesion in ADA-GEL. Recultured cells retained normal migration and proliferation, indicating that ADA-GEL can provide a more physiologically relevant 3D tumor microenvironment supporting cell attachment, spreading, and viability. This 3D in vitro tumor model provides a suitable platform for investigating diverse aspects of tumor cell behavior specific to colorectal cancer. On the other hand, it is known that cancer progression depends on interactions beyond ECM mimicry. Therefore, more investigations are needed to demonstrate that the model reproduces principal features of cancer (e.g., invasion, drug resistance, response to hypoxia). Future tests should demonstrate functional relevance, i.e., a truly predictive tumor model beyond enhanced cell adhesion and proliferation, and a realistic model with respect to colorectal cancer biology.

Another study reported the fabrication of semi-interpenetrating polymer network (semi-IPN) hydrogels based on collagen (C), polyurethane (PU), and maltodextrin (MD), aimed at evaluating the effect of varying MD content (0–50 wt%) on their physicochemical characteristics and biological behavior [228]. Increasing the MD fraction was associated with enhanced proliferation of immune and osteogenic cells alongside moderate inhibitory effects on colon cancer cell growth. Furthermore, these hydrogels, designed as bioactive scaffolds for both soft and hard tissue regeneration, immunomodulatory applications, and drug delivery, exhibited excellent hemocompatibility, antibacterial properties, and pH-responsive release profiles while also accelerating wound healing and supporting hydroxyapatite formation. Nonetheless, several unresolved features remain to be addressed. There are no data on long-term degradation or toxicity. Future investigations could show how the hydrogel balances pro-regenerative effects with anticancer cytotoxicity in the same biological environment. The anticancer effects of the material should also be evaluated in a combined model (e.g., wound, infection and cancerous environment) in order to demonstrate that all functions operate simultaneously without interference.

Advanced preclinical CRC models are essential for effective drug discovery, but most current 3D systems rely on non-human or synthetic materials that poorly mimic native tissue. In contrast, human (patient-derived) decellularized ECM hydrogels such as CologEM, designed as a biomimetic scaffold for advanced 3D modeling of colorectal cancer, preserve principal biochemical and structural features of the tumor microenvironment, thereby supporting tumor growth and drug response in a more physiologically relevant manner [229]. CologEM could be a promising platform for developing improved 3D CRC models, studying cancer progression and drug response, and advancing anticancer therapies. On the one hand, this innovative study links matrix properties to epithelial-mesenchymal transition (EMT) and drug resistance; on the other, however, there is a major unresolved feature, namely, how to reconcile the biological fidelity of patient-derived ECM (given the intrinsic variability of human biopsies depending on age, sex, and disease stage) with the need to maintain reproducibility and standardization in drug testing.

The primary treatment for melanoma remains surgical resection; however, controlling tumor recurrence while promoting effective wound healing continues to be a major postoperative challenge. To address the challenges of tumor recurrence and wound repair, a heterogeneous hybrid hydrogel scaffold was developed to enable sequential photothermal therapy and chemotherapy [230]. Using a 3D printing platform, an SA-GG@PDA hybrid hydrogel scaffold was fabricated from a composite bioink composed of sodium alginate (SA), gellan gum (GG), and different concentrations of polydopamine nanoparticles (PDA NPs). PDA NPs in the hybrid bioink enhanced the scaffold’s photothermal properties while the thermosensitive gelatin coating loaded with doxorubicin enabled photothermal-triggered drug release, providing combined photothermal and chemotherapeutic effects to inhibit tumor cell proliferation and recurrence post-surgery. The printed hybrid hydrogel scaffold with porous structure further promoted HUVEC proliferation, migration, and tissue ingrowth, supporting postoperative in vivo wound healing. In the same mouse model, sequential treatment using a heterogeneous SA-GG@PDA scaffold loaded with DOX was evaluated. The multifunctional SA-GG@PDA + DOX hydrogel scaffold showed potential for preventing tumor recurrence and enhancing in vivo wound healing post-surgery. This study addresses a real clinical problem, tumor elimination vs. tissue regeneration, and the sequential design is conceptually strong and adapted to the clinical case. Nevertheless, a critical aspect that warrants further investigation is the precise spatiotemporal regulation of therapeutic activity in order to rigorously determine whether the system can effectively delineate the transition between tumor eradication and subsequent tissue regeneration, both temporally and spatially. Furthermore, in conjunction with this need for precise spatiotemporal regulation, comprehensive long-term evaluations, including monitoring of tumor recurrence and the assessment of metastatic progression, are essential to fully validate the therapeutic efficacy and clinical relevance of the investigated system.

Osteosarcoma is a highly aggressive cancer that is the most common primary malignant bone tumor in adolescents and young adults, and remains associated with poor prognosis due to its aggressive behavior and early metastasis [231,232]. Its multifactorial origin includes genetic and environmental factors, and standard treatment relies on neoadjuvant chemotherapy followed by surgical resection [233]. Recent research has reported the development of 3D printed PLGA/PLA scaffolds functionalized with PEGylated gold nanobars and doxorubicin for dual-mode bone cancer therapy [234]. The study investigated the possibility of combined photothermal-chemotherapeutic treatment. Two 3D-printed scaffolds were developed: a PLGA/PLA system with GNRs@PEG-DOX (Target-1), and a variant coated with chitosan hydrogel (Target-2) which improved hydrophilicity and flexibility. Both enabled NIR-triggered drug release and effective photothermal therapy while significantly reducing osteosarcoma cell viability, highlighting their potential for localized treatment. FESEM analysis showed that all scaffolds had interconnected pores, with the control exhibiting larger pores (~700 μm), while gold nanorod incorporation (Target-1) reduced pore size (~400 μm) and increased surface roughness. Despite these changes, structural integrity and pore connectivity were preserved, with potential benefits for cell adhesion. This impressive study in multimodal therapy conceptually addresses both tumor eradication and bone defect repair, which is a real and critical clinical need in osteosarcoma. Unfortunately, there was no in vivo model of the osteosarcoma defect and no evaluation of tumor recurrence and bone healing under simultaneous treatment. In real clinical cases, residual tumor cells and regenerating bone cells coexist; therefore, the therapies cannot act independently. Thus, a central question to be addressed in future evaluations remains demonstrating whether the system can achieve selective cytotoxicity towards tumor cells while preserving and supporting normal bone-forming cells in the same scaffold environment without compromising them.

Table 6 provides a comparative overview of 3D/4D-printed hydrogel scaffolds and constructs applied in various the cancer therapies addressed in this section, summarizing their main characteristics, material composition, therapeutic strategies, underlying mechanisms, and associated biological outcomes.

## 6. Current Challenges and Limitations

While 3D and 4D printed hydrogel scaffolds hold significant promise for targeted drug delivery and wound healing within regenerative medicine thanks to their tunable architecture and dynamic responsiveness, several current challenges and limitations impede their broader implementation.

### 6.1. Mechanical Weakness and Structural Instability

One of the most persistent limitations of hydrogel-based scaffolds is their inherently poor mechanical strength and structural stability. While hydrogels are highly hydrated and biocompatible, their soft and fragile nature restricts their application in load-bearing tissues such as bone and cartilage. Even in soft tissue applications, insufficient mechanical integrity can lead to deformation, collapse, or premature failure during implantation and in vivo function. Although reinforcement strategies involving composite materials or nanofillers have been explored, achieving an optimal balance between mechanical robustness and biological functionality remains a significant challenge [23,235].

### 6.2. Limited Bioactivity and Incomplete Biomimicry

Despite advances in material design, many hydrogel systems, particularly synthetic ones, lack the intrinsic bioactivity required to fully replicate the native extracellular matrix (ECM). These materials often do not provide sufficient biochemical cues for cell adhesion, proliferation, and differentiation, resulting in suboptimal tissue integration. While natural hydrogels offer better bioactivity, they suffer from batch variability and limited tunability. Consequently, replicating the complex biochemical and structural features of native tissues continues to be a major obstacle in scaffold design [236,237].

### 6.3. Uncontrolled and Unpredictable Degradation Profiles

The degradation behavior of hydrogel scaffolds is a critical parameter that directly influences tissue regeneration outcomes. However, controlling degradation kinetics in vivo remains difficult due to variations in enzymatic activity, local pH, and immune responses. A mismatch between scaffold degradation and tissue formation can compromise healing: rapid degradation may lead to loss of structural support, whereas slow degradation can impede tissue remodeling and provoke chronic inflammation. Therefore, developing materials with predictable and tunable degradation profiles that align with specific clinical applications remains an ongoing challenge [49,53,238].

### 6.4. Bioink Limitations and Printability Constraints

The success of 3D bioprinting relies heavily on the rheological and physicochemical properties of bioinks. Achieving optimal printability requires a delicate balance between viscosity, shear-thinning behavior, and crosslinking kinetics. High-viscosity bioinks improve structural fidelity but can induce shear stress that damages encapsulated cells, while low-viscosity systems enhance cell viability but compromise shape retention. This trade-off limits the range of usable materials and complicates the fabrication of complex high-resolution structures [239,240,241].

### 6.5. Reduced Cell Viability and Functionality During Printing

Bioprinting processes can impose significant stress on living cells, leading to reduced viability and altered cellular function. Factors such as extrusion pressure, shear forces, temperature fluctuations, and exposure to ultraviolet light during photocrosslinking can negatively impact cell survival. Even when initial viability is maintained, subtle damage to cellular machinery may impair long-term proliferation and differentiation. Ensuring high cell viability while maintaining printing precision represents a critical bottleneck in the development of functional tissue constructs [242,243,244].

### 6.6. Insufficient Resolution and Architectural Control

Current bioprinting technologies are limited in their ability to achieve the fine spatial resolution required to accurately replicate the hierarchical architecture of native tissues. Control over pore size, interconnectivity, and microstructural features is often inadequate, which can negatively affect cell migration, nutrient diffusion, and drug release profiles. Although advanced techniques such as stereolithography and two-photon polymerization offer higher resolution, they are often constrained by material compatibility and scalability issues [207,245].

### 6.7. Vascularization and Mass Transport Limitations

The lack of effective vascularization remains one of the most critical challenges in the field. In thick or densely populated scaffolds, the diffusion of oxygen and nutrients is in-sufficient to sustain cell viability, leading to necrotic regions within the construct. While various strategies such as incorporating angiogenic factors or printing microchannels have been explored, the formation of stable and functional vascular networks that integrate with host tissue has still not been fully achieved. This limitation significantly restricts the clinical translation of large or complex engineered tissues [246].

### 6.8. Challenges in Controlled Drug Delivery

Although hydrogels have been widely investigated as drug delivery platforms, achieving precise control over drug release kinetics remains difficult. Factors such as heterogeneous network structures, diffusion limitations, and interactions between the drug and the polymer matrix can lead to unpredictable release profiles. Additionally, the efficient incorporation and sustained delivery of hydrophobic drugs poses significant challenges due to the inherently hydrophilic nature of most hydrogels. These limitations hinder the development of reliable and reproducible therapeutic systems [247].

### 6.9. Multifunctionality and System Integration Constraints

Designing scaffolds that simultaneously fulfill multiple roles, such as providing mechanical support, delivering drugs, and supporting cell growth, remains a complex task; integrating these functions into a single platform often results in trade-offs, where optimizing one property compromises another. For example, increasing mechanical strength may reduce porosity and impair cell infiltration, while enhancing drug loading capacity may affect structural stability. Achieving seamless integration of multiple functionalities without compromising overall performance is a major challenge [248,249].

### 6.10. Limitations in 4D Printing and Stimuli Responsiveness

While 4D printing introduces the possibility of dynamic and adaptive scaffolds, it also adds layers of complexity in material design and fabrication. Controlling the timing, extent, and reversibility of shape transformations or functional responses remains difficult, particularly in heterogeneous biological environments. Additionally, the long-term stability and biocompatibility of stimuli-responsive materials are not yet fully understood. These challenges limit the practical implementation of 4D systems in clinical settings [197].

### 6.11. Regulatory, Translational, and Scalability Barriers

Beyond technical challenges, significant barriers exist in the translation of hydrogel-based scaffolds from laboratory research to clinical practice. Regulatory approval processes are complex and time-consuming, requiring extensive validation of safety, efficacy, and reproducibility. Furthermore, the lack of standardized materials and fabrication protocols complicates quality control and large-scale manufacturing. High production costs and limited scalability further hinder commercialization, delaying the widespread adoption of these technologies in healthcare [143,250].

### 6.12. Lack of Long-Term In Vivo and Clinical Data

Finally, there is a notable scarcity of long-term in vivo and clinical studies evaluating the performance of 3D and 4D printed hydrogel scaffolds. Questions remain regarding their long-term biocompatibility, degradation byproducts, immune responses, and functional integration with host tissues. Without robust clinical evidence, it is difficult to fully assess the safety and efficacy of these systems, which in turn slows regulatory approval and clinical implementation [60,251].

Despite significant progress, 3D and 4D printed hydrogel scaffolds face substantial challenges that limit their clinical translation. Printability and structural stability remain primary technical hurdles; hydrogels often exhibit low mechanical strength and poor shape fidelity during and after printing, leading to collapse or deformation of complex geometries and difficulties in maintaining long-term integrity under physiological loads. Closely related are scalability and fabrication issues: transitioning from lab-scale constructs to clinically relevant sizes with consistent quality is constrained by slow printing speeds, limited resolution at larger scales, and challenges in integrating multiple materials or bioactive agents without compromising functionality. Bioprinting constraints such as bioink rheology, reduced cell viability, and limited resolution further hinder the fabrication of structurally and biologically optimal constructs. Pivotal issues include limited bioactivity and difficulty in achieving controlled degradation that matches tissue regeneration rates. In addition, insufficient vascularization and mass transport restrict the viability of large or complex tissues, while achieving precise and reproducible drug release remains problematic, particularly for hydrophobic therapeutics. The integration of multiple scaffold functions often introduces trade-offs, and 4D systems add further complexity in terms of responsiveness and stability.

Moreover, regulatory and safety concerns pose barriers to translation, as standard frameworks for evaluating bioprinted constructs are underdeveloped; ensuring reproducible performance, biocompatibility, and predictable degradation without adverse immune responses demands extensive preclinical validation that current regulatory pathways are not fully equipped to expedite. In addition, the lack of long-term clinical data impedes widespread adoption in regenerative medicine.

Economic factors also play a role. Costs and commercialization barriers are significant given the high expenses associated with advanced bioprinting hardware, specialized bioinks, and quality control systems.

Together, these technical, regulatory, and economic challenges underscore the need for interdisciplinary innovation to enable safe, effective, and scalable deployment of hydrogel-based printed scaffolds in clinical settings.

## 7. Future Perspectives and Emerging Trends

Future perspectives for 3D and 4D printed hydrogel scaffolds in regenerative medicine focus on personalized medicine and patient-specific implants, enabling constructs tailored to individual anatomical and pathological needs for optimized drug delivery and wound healing.

### 7.1. Advanced Stimuli-Responsive and 4D Hydrogel Systems

A major future direction in hydrogel-based scaffolds lies in the development of stimuli-responsive materials that enable dynamic adaptation to physiological environments. These so-called 4D printed systems are engineered to respond to external or internal triggers such as pH, temperature, enzymatic activity, light, or magnetic fields. Such responsiveness allows scaffolds to undergo controlled shape transformations or modulate drug release profiles in real time, thereby improving therapeutic precision. In wound healing, these systems can adapt to changing microenvironmental conditions (e.g., inflammation or infection), while in drug delivery they enable on-demand release kinetics. However, achieving precise and reproducible control over these transformations remains a crucial research focus, particularly in complex in vivo environments.

### 7.2. Biomimetic and Self-Assembling Hydrogel Architectures

Emerging strategies emphasize the design of biomimetic hydrogels that closely replicate the structure and function of the native extracellular matrix (ECM). Self-assembling peptide-based and supramolecular hydrogels are particularly promising, as they can form highly organized nanostructures that support cell adhesion, proliferation, and differentiation. These systems provide biochemical and mechanical cues that guide tissue regeneration more effectively than conventional hydrogels. Additionally, their tunable properties allow for precise control over degradation and bioactivity. Future research is expected to further integrate molecular-level design with bioprinting technologies to produce scaffolds with hierarchical organization across multiple length scales.

### 7.3. Multi-Material and Hybrid Scaffold Design

The integration of multiple materials within a single scaffold represents a critical advancement toward achieving multifunctionality. Hybrid scaffolds combining natural polymers (e.g., gelatin, alginate) with synthetic polymers (e.g., polyethylene glycol) or nanomaterials (e.g., graphene oxide, bioactive nanoparticles) can simultaneously address limitations in mechanical strength, bioactivity, and degradation control. Such multi-material systems also enable spatial patterning of different functionalities within the scaffold, allowing distinct regions to perform specialized roles such as structural support, drug delivery, or cell encapsulation. Continued progress in multi-nozzle and co-axial bioprinting technologies will be essential to fully realize the potential of these complex constructs.

### 7.4. Next-Generation Controlled Drug Delivery Platforms

Future hydrogel scaffolds are expected to evolve into highly sophisticated drug delivery platforms capable of precise spatiotemporal control. Innovations such as core–shell structures, layered architectures, and gradient-based designs allow for the sequential or simultaneous release of multiple therapeutic agents. These systems can be tailored to de-liver combinations of drugs, growth factors, or genetic material in a controlled manner, thereby enhancing therapeutic efficacy in wound healing and tissue regeneration. Additionally, the incorporation of responsive elements enables triggered release in response to specific physiological conditions, further improving targeting and reducing systemic side effects.

### 7.5. Vascularization and Perfusable Tissue Constructs

As one of the most critical challenges in tissue engineering, vascularization is also a major focus of future research. Advances in bioprinting techniques are enabling the fabrication of pre-vascularized scaffolds with embedded microchannel networks that facilitate nutrient and oxygen transport. Strategies such as the use of sacrificial bioinks, angiogenic growth factor incorporation, and co-culture with endothelial cells are being actively explored in order to promote rapid vascular integration in vivo. The development of perfusable constructs is particularly important for thick or complex tissues, where diffusion alone is insufficient to sustain cell viability.

### 7.6. Personalized and Patient-Specific Scaffold Fabrication

The convergence of bioprinting with medical imaging and computational modeling is paving the way for personalized regenerative therapies. Patient-specific scaffolds can be designed using data from imaging modalities such as CT or MRI, allowing for precise replication of anatomical defects. When combined with autologous cells, these customized constructs can significantly reduce the risk of immune rejection and improve clinical out-comes. Future developments are likely to focus on streamlining the workflow from imaging to fabrication, enabling rapid and cost-effective production of individualized scaffolds.

### 7.7. Integration with Stem Cell Therapy and Immunomodulation

Hydrogel scaffolds are increasingly being engineered to function as cell delivery vehicles, particularly for stem cells. By providing a supportive microenvironment, these scaffolds enhance cell survival, retention, and differentiation at the target site. Moreover, there is growing interest in designing scaffolds that actively modulate the immune response, promoting a regenerative rather than inflammatory environment. The combination of stem cell therapy with controlled drug delivery within a single platform represents a powerful approach for accelerating tissue repair and improving healing outcomes.

### 7.8. Artificial Intelligence and Computationally Guided Design

The application of artificial intelligence (AI) and machine learning in scaffold design is an emerging trend with transformative potential. AI-driven models can optimize scaffold architecture, predict material behavior, and simulate drug release kinetics, significantly reducing the need for trial-and-error experimentation. Computational tools also enable the design of complex geometries that would be difficult to achieve through conventional approaches. As data availability and computational power continue to grow, AI is expected to play an increasingly central role in accelerating innovation and translating laboratory discoveries into clinical applications.

### 7.9. Advances in Bioprinting Technologies

Technological innovations in bioprinting hardware and processes are expected to address many of the current limitations in scaffold fabrication. High-resolution printing techniques, multi-material deposition systems, and real-time monitoring technologies are improving the precision, reproducibility, and scalability of scaffold production. Additionally, developments in bioink formulation are enabling better control over printability and biological performance. These advances will facilitate the fabrication of more complex and functional tissue constructs, bringing the field closer to clinical and commercial viability.

### 7.10. Translation, Standardization, and Commercialization Pathways

In order for hydrogel-based scaffolds to achieve widespread clinical adoption, significant progress is required in standardization, regulatory approval, and scalable manufacturing. The development of standardized bioinks, reproducible fabrication protocols, and robust quality control measures will be essential for ensuring consistency and safety. At the same time, regulatory frameworks must evolve to accommodate the unique challenges posed by bioprinted and 4D materials. Efforts to reduce production costs and streamline manufacturing processes will further support commercialization, ultimately enabling broader access to these advanced therapeutic technologies.

Advances in clinical translation and real-world applications are increasingly bridging laboratory research with patient care, emphasizing scalable manufacturing, regulatory compliance, and cost-effective deployment with the aim of establishing these hydrogel-based scaffolds as practical tools for precision regenerative therapies.

## 8. Conclusions

Today, 3D and 4D printed hydrogel scaffolds are increasingly being investigated as platforms for targeted drug delivery and wound healing in regenerative medicine thanks to their tunable architecture and compatibility with biological systems. However, their performance remains constrained by limitations in printability, mechanical stability, reproducibility, and scalability as well as by challenges related to regulatory approval. In particular, the integration of stimuli-responsive functionality in 4D systems introduces additional complexity, including variability in response kinetics and long-term reliability.

While ongoing developments in biofabrication and material design continue to expand the capabilities of these systems, their clinical translation remains limited. Future work should focus on establishing robust structure–property relationships, improving multi-material and 4D printing strategies, and standardizing evaluation protocols. Greater emphasis on in vivo validation and clinically relevant models will be necessary to assess long-term performance and safety. Addressing these challenges will be critical in the development of reliable and scalable hydrogel-based therapies for regenerative applications.

## Figures and Tables

**Table 1:** Natural versus synthetic polymers used in the production of hydrogels.

Materials	Benefits	Drawbacks
Natural polymers	Controlled degradation rates, which can be designed to degrade slowly or on-demand [58] Wide chemical diversity, the final structure can be tailored with specific functional groups for targeted applications [59] Scalable production, easy to manufacture in large quantities with consistent quality [60] Thermal and chemical stability, often more resistant to heat, solvents, and harsh conditions than natural polymers [61] Enhanced shelf-life, typically more stable during storage [62] Cost-effective [63] Resemble the extracellular matrix, supporting cell attachment and proliferation [64] Typically sourced from renewable materials, eco-friendly [65] Intrinsic bioactivity, can interact with cells and tissues to promote healing or specific biological responses [66] Minimal toxicity, generally safe for medical and food applications [67] Flexible and soft, suitable for forming hydrogels, films, or scaffolds with desirable mechanical properties [68] Good water retention, can absorb and retain large amounts of water, useful in hydrogels [69] Easy enzymatic degradation allows controlled breakdown in biological environments [70] Versatile functional groups, natural chemical structures can be modified for crosslinking or drug delivery [71]	Limited mechanical strength [72] Variable quality, batch-to-batch consistency can be an issue [73] Rapid degradation, they may break down too quickly in some applications [74] Potential immunogenicity, some natural polymers can trigger immune responses [75] Processing challenges, difficult to modify or functionalize compared to synthetic polymers [76]
Synthetic polymers	Strong mechanical performance with adjustable properties [77] Reliable quality and reproducibility [78] Customizable for specific uses, including stimulus-responsive functions [79] Controlled degradation rates, which can be designed to degrade slowly or on-demand [80]. Wide chemical diversity, the final structure can be tailored with specific functional groups for targeted applications [81]. Scalable production, easy to manufacture in large quantities with consistent quality [82] Thermal and chemical stability, often more resistant to heat, solvents, and harsh conditions than natural polymers [83] Enhanced shelf-life, typically more stable during storage compared to natural polymers [84]. Cost-effective, production can be cheaper than isolating or extracting natural polymers [84]	Most of them have limited biocompatibility when used in medicine due to the adverse reactions they can cause in the body and persistence in the environment [85]. The majority are poorly biodegradable, many persist in the environment for many years, which raises concerns about disposal [86]. Potential toxicity, degradation products can sometimes be harmful [87] Complex synthesis, may require harsh chemicals or conditions [88] Less natural cell interaction, they often don’t mimic the extracellular matrix as effectively as natural polymers [89]

**Table 2:** Comparative analysis of natural, synthetic, and semi-synthetic hydrogels based on physicochemical and biological properties.

Property	Natural Hydrogels	Synthetic Hydrogels	Semi-Synthetic Hydrogels
Biocompatibility	Excellent	Moderate–high	High
Mechanical strength	Low (0.1–10 kPa)	High (10–1000 kPa)	Moderate (10–100 kPa)
Mesh size	Large (20–100 nm)	Small (5–20 nm)	Intermediate
Drug release	Fast (hours–days)	Slow (days–weeks)	Tunable
Degradation control	Limited	Precise	Tunable
Bioactivity	High	Low	Moderate–high

**Table 3:** Summary of recent hydrogel scaffold strategies in skin tissue engineering, highlighting their composition, functional properties, and effects on wound healing and tissue regeneration.

Study/Approach	Hydrogel Composition	Characteristics	Biological Function	Application Outcome
General biomimetic scaffolds and 3D bioprinting [187]	Hydrogels; dECM; bioinks	3D/4D tunability; biomimicry of ECM	Supports cell growth; metabolism; differentiation	Skin and musculoskeletal regeneration; personalized medicine
Stem cell; hydrogel systems [188]	Hydrogels; pluripotent stem cells	Patient-specific cells; low antigenicity	Enhances regeneration and integration	Improved wound healing potential
4D phototunable hydrogels [189]	Photochemically regulated hydrogels	Spatiotemporal control; dynamic ECM mimicry	Controls proliferation; migration; differentiation	Advanced tissue modeling; drug screening; organoid stability
P-coumaric acid (P-CA) scaffold [190]	Chitosan, PEO, PCL, P-CA	Sustained drug release; printable scaffold	Antioxidant; antibacterial effects	Enhanced wound healing and infection control
Hyaluronic acid, dopamine hydrogel [191]	Enzyme-crosslinked HA, (dopamine-modified)	Self-healing; adhesive; porous structure	Supports BMSCs; promotes tissue regeneration	Accelerated healing; hair follicle formation; minimal scarring
3D-printed hydrogel scaffold incorporating FLA@ZIF-8 nanoparticles [192]	κ-carrageenan, konjac glucomannan, flavanone-loaded nanoparticles	Dual-network, controlled Zn^2+^; drug release	Antibacterial; promotes cell proliferation	Effective healing of infected wounds
Zn/tannic acid chitosan hydrogel [193]	Glycol chitosan, Zn, tannic acid	Strong mechanical properties; immunomodulatory	Anti-inflammatory (M2 macrophage polarization)	Faster regeneration; collagen deposition; re-epithelialization
4D-printed hydrogel; gene therapy [194]	4D-printed hydrogel; piRNA antagomir	Controlled microenvironment; gene regulation	Enhances stem cell survival; migration; angiogenesis	Improved diabetic wound healing

**Table 4:** An overview of hydrogel-based and 3/4D-printed scaffold strategies for minimally invasive implantation, with focus on materials, deployment approaches, and biomedical applications.

Study/Approach	Material/Technology	Main Characteristics	Minimally Invasive Strategy	Application Outcome
3D and 4D printing technologies for smarter biomedical approaches [195]	Smart materials; AI-assisted 4D printing	Shape-morphing; adaptive; in situ fabrication	Direct in situ printing on target tissues	Improved fit to complex anatomy; high-fidelity biomedical constructs
In situ patterning of the hydrogel in vivo for minimally invasive implantation [196]	Injectable hydrogels; enzymatic crosslinking	Soft, biocompatible; tunable crosslinking	Injection-based patterning without open surgery	Implantable scaffolds and devices formed directly in the body
General 4D bioprinting applications [197]	Stimuli-responsive hydrogels	Dynamic response to stimuli; multifunctionality	Deployment in compact form, activation after implantation	Devices such as stents, scaffolds, and wound closures
Adaptive 4D biomaterials in medicine [198]	Hydrogels, shape-memory polymers	Time-dependent transformation; spatiotemporal control	Minimally invasive insertion followed by in vivo transformation	Personalized implants; controlled drug delivery; regenerative scaffolds
Advanced AM (3D–6D printing) [199]	Hydrogels; multi-axis additive manufacturing	Increased complexity; strength; biomimicry	Smaller initial structures transformed post-implantation	Custom implants and scaffolds for tissue engineering
4D thermoset polyurethane scaffolds [200]	Amphiphilic shape-memory polyurethanes	Shape memory; swelling-induced morphing; stiffness transition	Transcatheter delivery as compact structure; expands in vivo	Void-filling scaffolds with programmable 3D architecture

**Table 5:** Summary of the main characteristics of advanced 3D/4D printed hydrogel-based scaffolds for bone regenerative medicine.

Study/Scaffold Type	Materials/Composition	Printing/Fabrication Method	Main Functional Features	Mechanical Properties	Drug/Bioactive Delivery	Biological Outcomes
pH-responsive HA–PSCPP scaffold [202]	Hydroxyapatite, PSCPP hydrogel (polyacrylic acid, sodium alginate, CMC, potato starch, propyl gallate), curcumin	Porous scaffold fabrication	pH-responsive swelling; >70% porosity	Improved vs. HA alone	Dual-phase release: fast at pH 5.4 (90%), slow at pH 7.4 (3%/7 days)	>80% cell viability
TONC-reinforced alginate scaffold [204]	Sodium alginate; TEMPO-oxidized nanocellulose (TOCNFs/TOCNCs from coffee waste)	Extrusion-based 3D printing	Enhanced rheology; printability; swelling	Young’s modulus ~227 kPa	Diffusion-controlled release	Excellent biocompatibility and proliferation
Enzyme-responsive honeycomb scaffold [206]	PLGA/β-TCP; icaritin (outer), hyaluronic acid hydrogel, vancomycin (core)	Low-temp 3D printing	Enzyme-triggered (hyaluronidase) response	Elastic modulus ~0.8 MPa	On-demand antibiotic release	Eliminates MRSA; promotes BMSC osteogenesis
Fibrous scaffold; injected hydrogel [208]	PCL micro/nanofibers, hyaluronan hydrogel (cell-loaded)	Meltblown; electrospinning; injection bioprinting	High porosity; hydrophilic environment	Good structural stability	Cell delivery	Enhanced osteoblast proliferation
rGO/GelMA dual-functional scaffold [209]	Reduced graphene oxide, GelMA, SCs, BMSCs	3D printing	Neurogenic; osteogenic differentiation	Improved compressive strength (rGO-dependent)	Cell-based (no drug release)	~85% viability; nerve; bone regeneration

**Table 6:** Summary of principal features of 3D/4D printed hydrogel scaffolds or constructs used in cancer therapy.

Main Characteristics	Material/Composition	Drug/Therapeutic Strategy	Function/Mechanism	Biological Outcomes	Cancer Type
Dual-purpose scaffold for drug delivery and tissue support [222]	Methacrylate-modified chitosan, methylcellulose	Doxorubicin	Controlled drug release; local therapy	Rejuvenated rBMSC viability; potential recurrence inhibition	Melanoma
ECM–ion channel interactions studied via hydrogel modeling [226]	Not directly hydrogel-based; focus on ECM and signaling	N/A	Mechanistic modeling of tumor-stroma crosstalk	Identified therapeutic targets related to ECM remodeling	Colorectal Cancer
3D cell culture model mimicking ECM [227]	Pure alginate vs. cross-linked alginate–gelatin (ADA-GEL)	N/A	Supports adhesion, spreading, and proliferation	High proliferation; cellular network formation; physiological cell behavior	Colorectal Cancer
Semi-IPN hydrogel with multifunctional properties [228]	Collagen, Polyurethane, Maltodextrin	Ketorolac, methylene blue	pH-sensitive release, tissue regeneration, immunomodulation	Promoted metabolic activity of healthy cells, selective cytotoxicity to cancer cells	Colon Cancer
Patient-derived ECM hydrogel for 3D tumor model [229]	Decellularized colon ECM (CologEM)	N/A	Mimics tumor microenvironment, supports drug testing	Biocompatibility, induces mesenchymal phenotype; antitumor drug resistance	Colorectal Cancer
Heterogeneous hybrid hydrogel for sequential therapy [230]	Sodium alginate, gellan gum, polydopamine, gelatin	Doxorubicin	Photothermal-triggered chemotherapy	Reduced tumor proliferation; enhanced wound healing	Melanoma
3D-printed scaffold with nanorods for combined therapy [234]	PLGA/PLA, PEGylated gold nanorods (GNRs@PEG), chitosan coating	Doxorubicin	Photothermal; chemotherapy	Reduced MG63 osteosarcoma viability < 20%, on-demand drug release	Osteosarcoma

## Data Availability

No new data were created or analyzed in this study.
